# Slr4, a newly identified S‐layer protein from marine Gammaproteobacteria, is a major biofilm matrix component

**DOI:** 10.1111/mmi.14588

**Published:** 2020-09-15

**Authors:** Sura Ali, Benjamin Jenkins, Jiujun Cheng, Briallen Lobb, Xin Wei, Suhelen Egan, Trevor C. Charles, Brendan J. McConkey, John Austin, Andrew C. Doxey

**Affiliations:** ^1^ Department of Biology University of Waterloo Waterloo ON Canada; ^2^ Metagenom Bio Life Science Inc. Waterloo ON Canada; ^3^ Centre for Marine Science and Innovation and School of Biological, Earth and Environmental Sciences The University of New South Wales Sydney Sydney NSW Australia; ^4^ Bureau of Microbial Hazards Health Products and Food Branch, Health Canada Ottawa ON Canada

**Keywords:** biofilms, extracellular matrix, marine bacteria, *Pseudoalteromonas tunicata*, S‐layer

## Abstract

S‐layers are paracrystalline proteinaceous lattices that surround prokaryotic cells, forming a critical interface between the cells and their extracellular environment. Here, we report the discovery of a novel S‐layer protein present in the Gram‐negative marine organism, *Pseudoalteromonas tunicata* D2. An uncharacterized protein (EAR28894) was identified as the most abundant protein in planktonic cultures and biofilms. Bioinformatic methods predicted a beta‐helical structure for EAR28894 similar to the *Caulobacter* S‐layer protein, RsaA, despite sharing less than 20% sequence identity. Transmission electron microscopy revealed that purified EAR28894 protein assembled into paracrystalline sheets with a unique square lattice symmetry and a unit cell spacing of ~9.1 nm. An S‐layer was found surrounding the outer membrane in wild‐type cells and completely removed from cells in an EAR28894 deletion mutant. S‐layer material also appeared to be “shed” from wild‐type cells and was highly abundant in the extracellular matrix where it is associated with outer membrane vesicles and other matrix components. EAR28894 and its homologs form a new family of S‐layer proteins that are widely distributed in Gammaproteobacteria including species of *Pseudoalteromonas* and *Vibrio*, and found exclusively in marine metagenomes. We propose the name Slr4 for this novel protein family.

## Graphical abstract

The S‐layer is the outermost protein layer surrounding bacterial cells. We discovered a new family of S‐layer proteins in marine bacteria that forms a paracrystalline square lattice surrounding cells. In addition, the S‐layer was found surrounding extracellular components in biofilms, suggesting that it may provide physical protection not only to cells but also to extracellular structures.

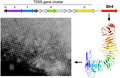

## 1 INTRODUCTION

S‐layers are self‐assembling, paracrystalline protein layers that form a two‐dimensional array on the surface of many bacterial and archaeal species. Ubiquitous across a large number of bacteria and archaeal phyla, S‐layer proteins can constitute 10% of the proteome of prokaryotic cells, making them one of the most abundant protein polymers found on earth (Qing, 2017). This combined with their diverse functional roles, which include the regulated nutrient transport through porous channels, physical protection of cells against the extracellular environment, targeted cell‐adhesion, biofilm interactions, and others, make S‐layers one of the most important cellular structures in the microbial biosphere (Beveridge *et al*., 1997; Sára and Sleytr, 2000; Fagan and Fairweather, 2014; Sleytr *et al*., 2014).

S‐layers have been extensively characterized in some bacterial species including *Caulobacter crescentus*, *Bacillus anthracis*, *Clostridiodes difficile*, and *Campylobacter fetus*. Electron microscopy (EM) and electron cryotomography studies of S‐layers (Bharat *et al*., 2017) have revealed their exquisite nanoscale organization into paracrystalline arrays formed typically by a single protein or glycoprotein subunits. Depending on inter‐subunit interactions between the S‐layer protein, S‐layer arrays can form different geometries, which include oblique (p1, p2), square (p4), or hexagonal (p3, p6) lattice symmetries (Sára and Sleytr, 2000). Interunit spacing within S‐layer lattices can range widely from 4 to 35 nm, depending on the structure of the S‐layer subunit and interactions between the subunits (Sleytr *et al*., 2014). Another important feature of S‐layer lattices is the presence of pores, which facilitate selective uptake of small molecular weight nutrients and potentially larger compounds in and out of the cell (Arbing *et al*., 2012). A prime example of S‐layer molecular architecture is the RsaA S‐layer protein from *C. crescentus*. RsaA forms a hexameric two‐dimensional array that is stabilized by Ca^2+^ ions and multiple protein‐protein interfaces. The RsaA S‐layer lattice contains pore sizes ranging from ~20 to 27 Å wide, thus forming a barrier for extracellular attack by phages and most macromolecules (Bharat *et al*., 2017).

Both Gram‐negative and Gram‐positive bacteria may possess S‐layers as the outermost layer of the cell. In Gram‐negative species, S‐layers are attached to the outer cell membrane through interactions with surface molecules such as lipopolysaccharide (von Kügelgen *et al*., 2020). In Gram‐positive organisms, S‐layers are attached to the secondary cell wall polymers or peptidoglycan through phosphodiester bonds or lipid anchors (Sára, 2001; Schuster and Sleytr, 2014). There are several families of protein domains/motifs that have been shown to mediate S‐layer anchoring to the outer cell layers. S‐layer proteins of some Firmicutes species contain S‐layer‐homologous (SLH) motifs within the N‐terminal region, which have been shown to mediate binding to the cell envelope (Mesnage *et al*., 2000). In other organisms (e.g., *C. difficile* and other clostridia), CWB2 motifs have been identified as cell wall anchoring modules (Willing *et al*., 2015). These anchoring domains have not been identified in all S‐layer proteins (e.g., those from Gram‐negative organisms), suggesting distinct cell envelope anchoring mechanisms.

In order to assemble S‐layers, bacteria and archaea must be able to secrete efficiently large quantities of S‐layer proteins across the cell envelope. For instance, it has been estimated that *C. difficile* must secrete ~400 S‐layer subunits per second to build a contiguous S‐layer composed of ~500,000 subunits (Fagan and Fairweather, 2014). In *C. difficile* as well as *B. anthracis*, S‐layer secretion is accomplished through the accessory sec system involving the accessory ATPase SecA2 and a pore containing SecYEG (*C. difficile*) or Sec2YEG (*B. anthracis*). In *Aeromonas*, S‐layer proteins are secreted through a dedicated type II secretion system involving a secretion apparatus similar to that of type IV pili (Tomás, 2012). In *Caulobacter* and several other Gram‐negative species, S‐layer secretion is accomplished through the type I secretion system, which consists of an outer‐membrane pore and inner membrane ABC transporter (Awram and Smit, 1998). In *C. crescentus*, these components are genetically encoded immediately upstream of the *rsaA* gene (Awram and Smit, 1998).

Despite their ubiquity across bacteria and archaea, the S‐layer proteins of many important lineages of bacteria remain uncharacterized. For example, *Pseudoalteromonas*, an ecologically significant genus of marine Gammaproteobacteria associated with marine eukaryotes and biofilm colonization (Bowman, 2007), has not been fully characterized in terms of its surface layer proteins. *Pseudoalteromonas tunicata,* originally isolated from tunicates (Holmstrom *et al*., 1998; Holmström and Kjelleberg, 1999) has become a model organism for studying eukaryotic host‐bacteria interactions in marine environments, and surface‐associated lifestyles (Thomas *et al*., 2008). *P. tunicata* is noteworthy for its “antifouling” activities and ability to colonize mixed species biofilms, where it secretes molecules to inhibit the growth of competing species (Holmström *et al*., 2002; Rao *et al*., 2005; 2010). *P. tunicata* exhibits broad antimicrobial capabilities, and produces antifungal (Franks *et al*., 2006), anti‐nematode (Ballestriero *et al*., 2010), and algicidal molecules (Lovejoy *et al*., 1998; Egan *et al*., 2001). In addition, *P*. *tunicata* possesses homologs of virulence factors that aid in host surface colonization (Gardiner *et al*., 2014; Eckhard *et al*., 2017). Characterization of *P. tunicata*, its extracellular proteome, and biofilm development is thus important to gain insights into the molecular mechanisms and physiology of marine biofilm development (Mai‐Prochnow *et al*., 2004; Rao *et al*., 2005).

In this study, we report the bioinformatics‐aided discovery and characterization of a new S‐layer protein from the marine organism *Pseudoalteromonas tunicata* and related species. We identify EAR28894 as the most abundant protein produced by *P. tunicata* planktonic and biofilm cultures, and predict its function as an S‐layer protein using structure prediction. We then characterize the lattice ultrastructure formed by Slr4, and investigate its presence on cells and in biofilms. We demonstrate that Slr4 forms a new family of S‐layer proteins predominantly in marine Gammaproteobacteria, which likely plays an important role in marine microbial physiology and ecology by functioning as a key structural constituent of biofilms.

## 2 RESULTS

### 2.1 Identification of an abundant protein in cultures of *Pseudoalteromonas tunicata*

**FIGURE 1:**
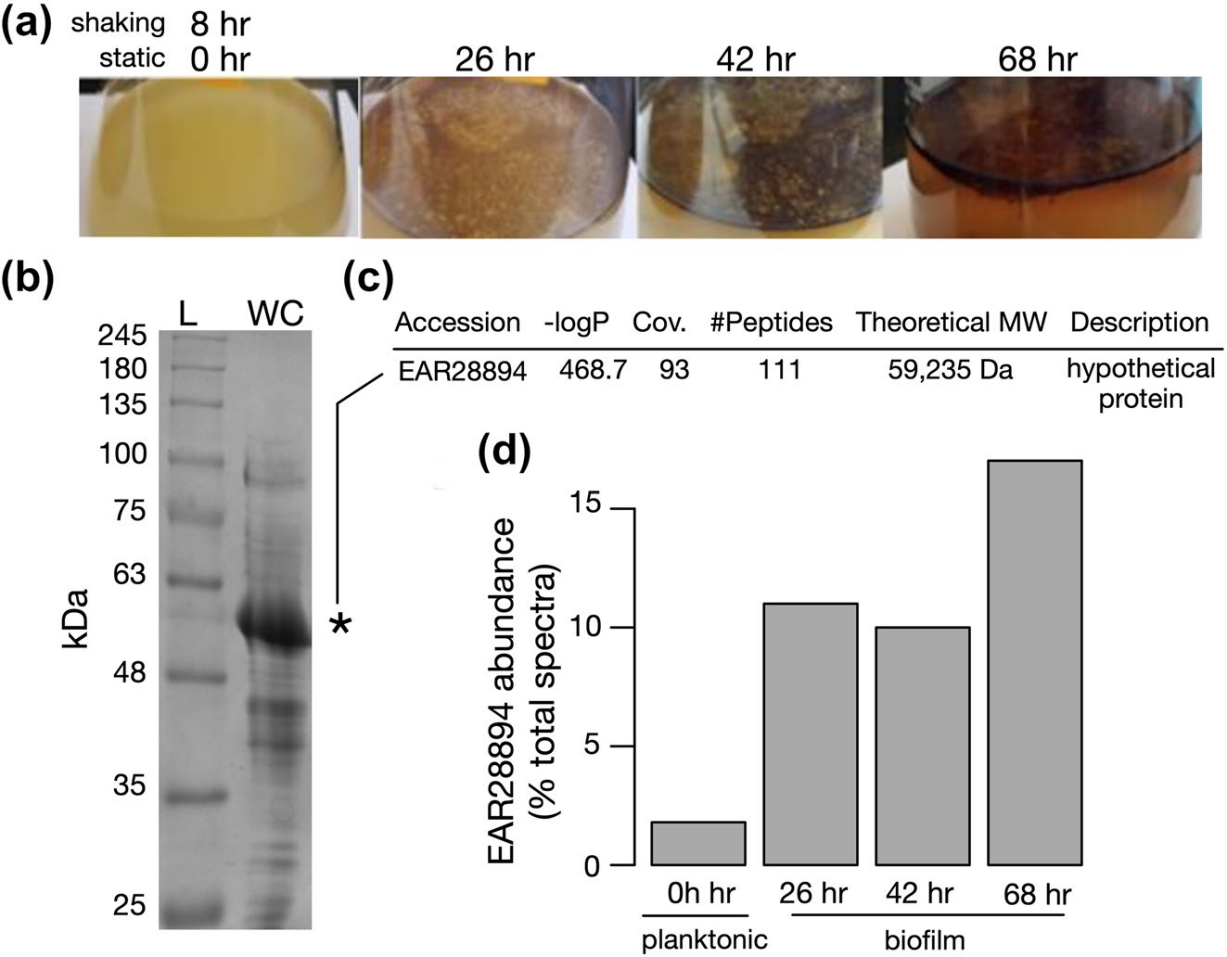
Identification of an abundant hypothetical protein in the *Pseudoalteromonas tunicata* proteome. (a) Liquid cultures of *P. tunicata* grown in Difco marine broth for 8 hr with shaking, followed by 26, 42, and 68 hr of static growth (no shaking) to induce pellicle biofilms. (b) SDS‐PAGE analysis of whole cell extract from planktonic cultures revealed a dominant band at ~57 kDa. L – ladder; WC – whole cell extract. (c) The ~57 kDa protein was identified by LC–MS/MS as a “hypothetical protein” EAR28894. (d) Shotgun proteomic analysis by LC–MS/MS of *P. tunicata* planktonic and biofilm samples confirmed EAR28894 as the most abundant expressed protein, constituting ~2% of total spectra in planktonic cultures and 10%–17% of total spectra in biofilms

*Pseudoalteromonas tunicata* D2 liquid cultures were initially grown in Difco Marine broth for 8 hr under shaking conditions (Figure 1a). Further incubation under static (non‐shaking) conditions for 26, 42, and 68 hr produced visible pellicle biofilms that formed at the air‐liquid interface with increasing thickness and biomass observed over time (Figure 1a). SDS‐PAGE analysis of total extracted protein at all stages revealed a dominant band of ~57 kDa (see Figure 1b for a representative gel). This protein band was identified by liquid chromatography–tandem mass spectrometry (LC–MS/MS) with spectra matching a ~59 kDa “hypothetical protein” (EAR28894, PTD2_07610) from *P. tunicata* D2 (Figure 1c). The LC–MS/MS identification was of high quality with 93% sequence coverage (Figure 1c, Tables S1 and S2). A BLAST search of EAR28894 failed to identify any homologs of known function; all significant matches (*E* < 0.001) were hypothetical proteins. No domains were detected using either the NCBI Conserved Domain Database (Marchler‐Bauer *et al*., 2015) or Pfam (Finn *et al*., 2014).

Shotgun LC–MS/MS analysis of total protein derived from 8 hr (shaking), 26–68 hr (static) cultures confirmed hypothetical protein EAR28894 as the most abundant protein at all time points (Data File S1). In planktonic cultures, the hypothetical protein EAR28894 was the most abundant protein and comprised 1.8% of total spectra followed by EF‐Tu (EAR26375) at 1.3%. In pellicle biofilms, the relative abundance of EAR28894 peptides increased markedly to 10%–17% of total spectra (Figure 1d). The second most abundant identified protein spectra in biofilms was EAR28646, a TonB‐dependent receptor, present at 2.1%–2.6% of total spectra. It is important to note that, although EAR28894 was the most abundant protein according to the percentage of total peptide spectra, these values do not necessarily reflect an accurate measurement of its true protein abundance.

Analysis of MS spectra mapping to the EAR28894 protein sequence revealed coverage across the full‐length protein, with the exception of an N‐terminal segment of 13–27 residues in length which was absent in all four data sets (Figure S1). This suggests that EAR28894 is proteolytically processed to remove its N‐terminal signal peptide, resulting in a mature protein on the gel (~57 kDa). Analysis of the MS/MS data also revealed an extreme abundance of non‐tryptic peptides associated with EAR28894 (Figure S2), indicative of extensive proteolytic processing.

### 2.2 Protein structure prediction suggests EAR28894 is an S‐layer protein

**FIGURE 2:**
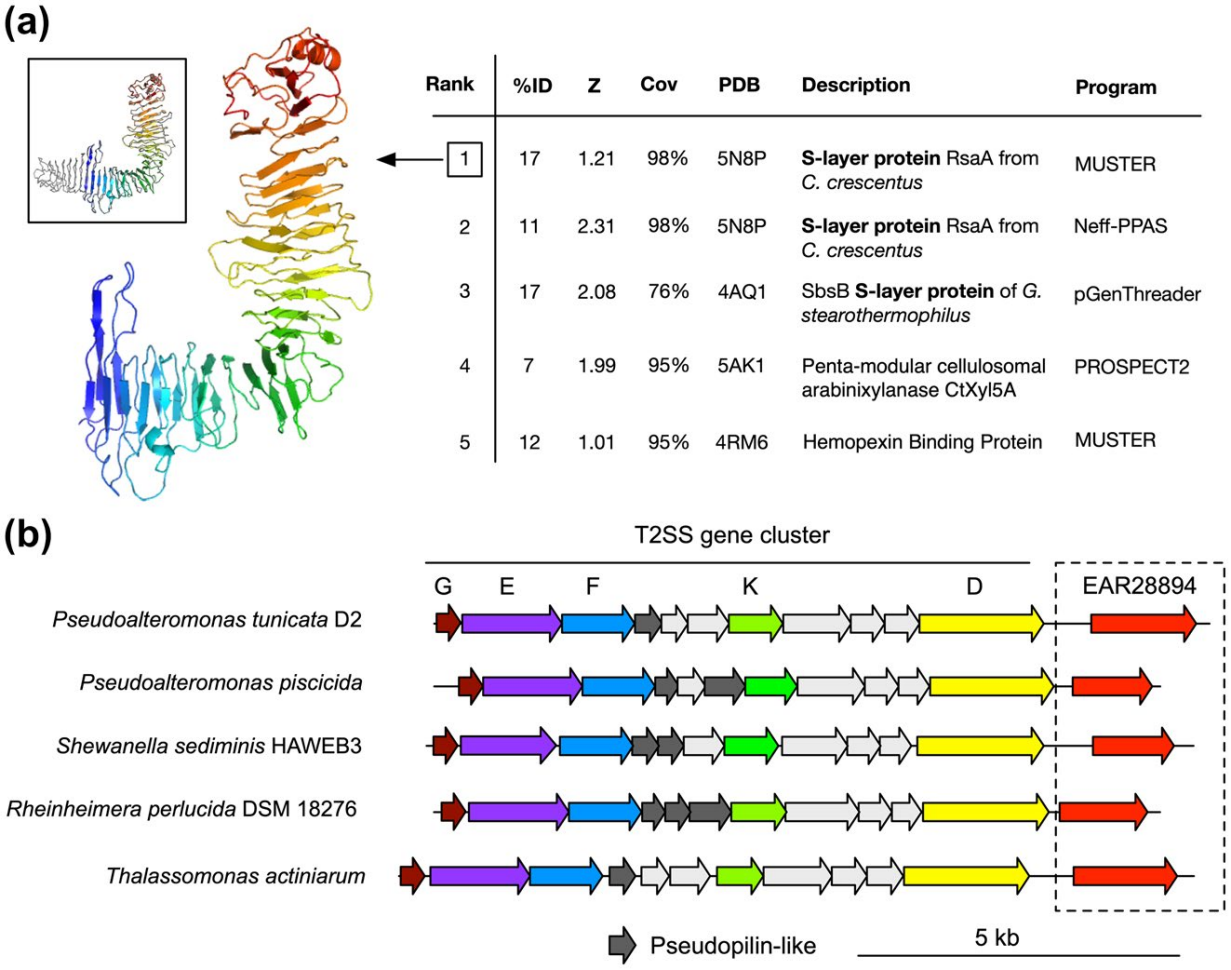
Bioinformatic analysis predicts *P. tunicata* EAR28894 hypothetical protein as an S‐layer protein. (a) Structure prediction by I‐TASSER. The top five threading templates are listed on the right, and the structural model for top‐ranked template is shown on the left. EAR28894 threaded onto the structure of *Caulobacter* RsaA (PDB ID 5n8p) as shown in the top left inset box with the template colored in gray. (b) Genomic context surrounding the EAR28894 gene in *P. tunicata* D2 and related genes in other genomes. Immediately upstream of the EAR28894 gene is a type II secretion gene cluster, suggesting a possible dedicated operon for type II S‐layer secretion

To predict the structure and function of the EAR28894 protein, we applied the I‐TASSER pipeline (Yang *et al*., 2014). I‐TASSER predicted an L‐shaped, almost entirely beta‐helical fold, and identified the crystal structure of *Caulobacter* S‐layer protein RsaA (PDB ID 5n8p) as the top structure template with high estimated model quality (low root‐mean squared deviation of 0.98 Å and high model coverage of 98%) (Figure 2a). Due to the repetitive and beta‐rich composition of RsaA, EAR28894 threaded onto different regions of the RsaA structure, resulting in two different high‐scoring alignments of only 11%–17% identity (ranked 1 and 2 in Figure 2a). Therefore, although a general L‐shaped beta‐helical fold and S‐layer structure/function can be predicted for EAR28894, its relationship to RsaA is unclear.

In addition, we examined the genomic context of EAR28894 in *P. tunicata* to determine whether additional evidence could be found supporting its prediction as an S‐layer protein. We observed that the EAR28894 gene is located immediately downstream of a putative type II secretion (T2S) pathway operon encoding several T2S genes (*T2SG*, *E*, *F*, *K*, *D*, and others), also known as general secretory pathway *gsp* genes (Figure 2b). Similar genomic arrangements were identified surrounding homologous genes identified in the genomes of other species (Figure 2b). This suggests the possibility of an evolutionarily conserved operon for EAR28894 secretion by a dedicated T2SS. Also consistent with T2S, signal peptide prediction by Signal P 5.0 predicted a Sec/SPI signal peptide with 98.7% likelihood and a cleavage site at position 23‐24 with 89.4% probability, which coincides closely with the absent N‐terminal region identified by MS (Figure S1). Notably, type II (*sec*‐dependent) secretion pathways have been identified as a dominant secretion mechanism for other bacterial S‐layer proteins (Boot and Pouwels, 1996).

### 2.3 Confirmation of EAR28894 as an S‐layer protein using TEM and knockout studies

**FIGURE 3:**
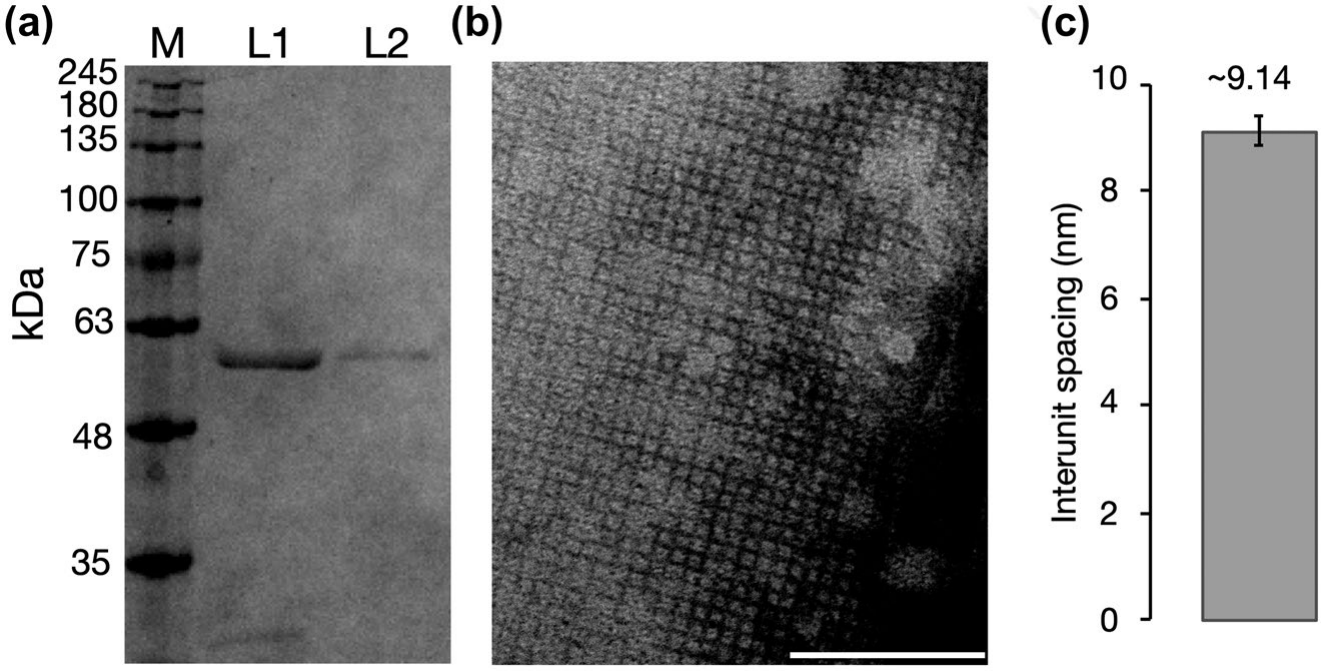
TEM imaging of purified EAR28894 reveals a paracrystalline lattice with square (p4) symmetry. (a) Purification of EAR28894 by ultracentrifugation (L1) followed by detergent washes (L2). L1 samples predominantly contain the ~57 kDa band (EAR28894) but also contained other impurities. L1 was, therefore, further purified using RIPA detergent washes to L2, which produced a single band on the gel. (b) TEM imaging reveals a paracrystalline lattice with a square (p4) symmetry. There are approx. 11 subunits per 100 nm, suggesting an inter‐unit spacing of approx. 9.1 nm. (c) Quantitative estimate of inter‐unit spacing based on averages of N = 5 independent measurements from TEM images. Scale bars = 100 nm

Transmission electron microscopy (TEM) was used to investigate the predicted S‐layer structure and function of EAR28894. EAR28894 was isolated and purified using differential centrifugation followed by a detergent wash with radioimmunoprecipitation assay (RIPA) buffer (see, Methods for details) (Figure 3a). TEM analysis of purified EAR28894 samples revealed S‐layer material characterized by a square lattice (p4) symmetry (Figure 3b). Based on repeated measurements using imageJ (5 independent replicates), the unit cell spacing was determined to be ~9.14 nm ± 0.27 (Figure 3c). This is consistent with the expected range of an S‐layer unit cell (4 to 35 nm). We, therefore, designated EAR28894 as “Slr4” given its identification as an S‐layer protein producing a fourfold symmetric lattice.

**FIGURE 4:**
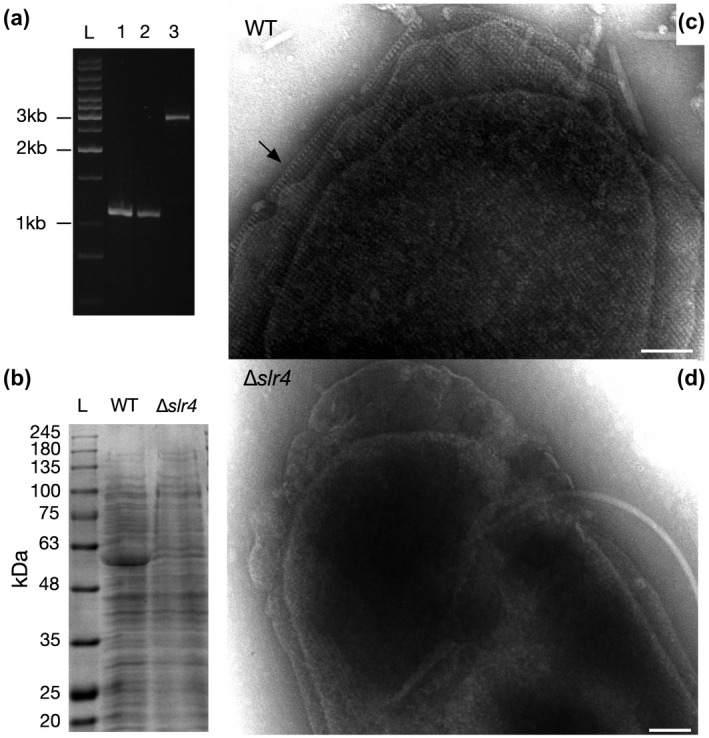
Electron micrographs of *P. tunicata* revealing presence/absence of a square grid S‐layer in WT and Δ*slr4* cells, respectively. (a) Verification of Δ*slr4* (EAR28894 gene deletion) mutant in *P. tunicata* PnAD3. PCR amplification of the PnAD3 genomic DNA with oligo JC470 and JC492 yielded a DNA fragment of same size (1,086 bp) as that from plasmid pJC272, but a 2814‐bp product was obtained from wild type (WT) DNA. Lane L, DNA marker; lane 1, *P. tunicata* PnAD3; lane 2, plasmid pJC272; lane 3, *P. tunicata* WT D2. (b) SDS‐PAGE analysis of whole cell extract of WT versus Δ*slr4* strains. (c) TEM micrographs of WT vs (d) Δ*slr4* cells, revealing the presence and absence of an S‐layer, respectively. A side‐view of S‐layer revealing U‐shaped subunits is indicated by an arrow in (c). Samples were derived from liquid cultures grown in marine Difco broth for 8 hr in shaking conditions. Scale bars = 100 nm

Using homologous recombination, we generated a Δ*slr4* deletion mutant. The coding region (1,737 bp) of the *slr4* gene (EAR28894) was replaced with a PstI restriction enzyme site (CTGCAG), which was verified by PCR amplification (Figure 4a) and Sanger sequencing. The Δ*slr4* mutant was also confirmed by SDS‐PAGE, which demonstrated a lack of the ~57 kDa band identified previously as EAR28894 (Slr4) (Figure 4b). The Δ*slr4* mutant strain was capable of growth in marine media (liquid and plates), formed colonies with similar morphology to the WT, and formed pellicle biofilms with similar appearance to the WT (Figure S3).

*P. tunicata* WT and Δ*slr4* cells were then visualized and compared by TEM. In untreated samples, S‐layers were difficult to observe on WT cells due to the presence of an apparent outer capsular polysaccharide layer (Figure S4); indeed, a capsular polysaccharide gene operon is present in the *P. tunicata* genome (Thomas *et al*., 2008) and has been identified in closely related species (Zeng *et al*., 2019). We, therefore, treated the cells with 0.1M Tris–HCl and a tissue homogenizer to remove the capsular layer and reveal the underlying S‐layer, similar to that done previously for the *Bacillus anthracis* S‐layer (Mesnage *et al*., 1998). In TEM micrographs of treated WT cells, an S‐layer lattice was observed completely surrounding the outer membrane of intact *P. tunicata* cells (Figure 4c) with an identical square lattice structure and geometry to that observed in purified Slr4 samples (Figure 3). Visualization of cells also revealed a “side view” of the S‐layer edge consisting of “V” shaped subunits (see arrow in Figure 4c). As predicted, the paracrystalline S‐layer was completely absent in TEM micrographs of Δ*slr4* mutants (Figure 4d), confirming that Δ*slr4* is required for assembly of an S‐layer in native cells.

### 2.4 S‐layer is shed from cells and is a major component of the biofilm matrix

**FIGURE 5:**
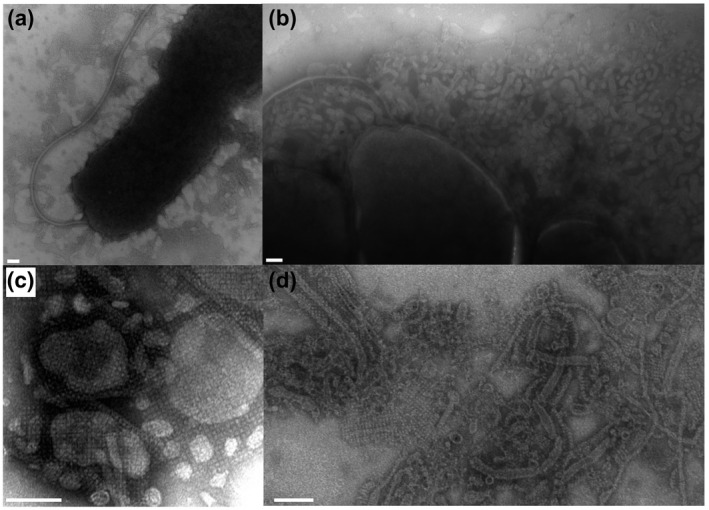
S‐layer is present in the *P. tunicata* extracellular environment and biofilm matrix. (a) A TEM micrograph showing a *P. tunicata* cell as well as “shed” extracellular material layer that is nearby but distinct from the cell body. (b) Three adjacent cells within a microcolony with a substantial amount of secreted outer membrane vesicles (OMVs) and a sheathed flagellum. (c) TEM micrograph of an extracellular protein fraction showing S‐layer associated with outer membrane vesicles of various forms. (d) TEM image taken from a WT pellicle biofilm, which shows S‐layer material in association with outer membrane vesicles, and fibrous and tubular structures. (e.g., see high‐magnification image shown in d) representing possible pili, flagella, prosthecae, or flagellar sheaths. Scale bars = 100 nm

Exclusively in the WT S‐layer producing strain, we observed an abundance of extracellular material containing S‐layer that appeared to be “shed” from the cell (Figure 5), as described in other organisms (Schultze‐Lam *et al*., 1992; Chandramohan *et al*., 2018). Extracellular matrix containing material resembling S‐layer was observed outside of planktonic cells (Figure 5a), as well as surrounding microcolonies of cells in biofilms (Figure 5b). The biofilm extracellular matrix also contained outer membrane vesicles (OMVs), tubular structures, and filaments (Figure 5b). Closer examination of purified extracellular matrix revealed S‐layer associated with outer‐membrane vesicles, (Figure 5c), and surrounding networks of filamentous structures including putative flagella, flagellar sheaths, pili, and prosthecae (Beurmann *et al*., 2017) (Figure 5d). S‐layer coated vesicles have been reported in archaea, where the S‐layer may provide physical stabilization at extreme conditions (Marguet *et al*., 2013; Rodrigues‐Oliveira *et al*., 2017). TEM imaging of WT 48 hr pellicle biofilms also revealed dense clusters of cells that were interconnected by S‐layer‐associated biofilm matrix components (Figure S5). TEM micrographs of Δ*slr4* mutant biofilms revealed cell clusters connected by biofilm matrix components but without any S‐layer material as expected (Figure S5). Δ*slr4* mutant biofilms also contained cells with deformed cell shapes (Figure S5), consistent with previous studies demonstrating the involvement of S‐layers in cell‐shape determination (Poppinga *et al*., 2012). These observations together with the high abundance of Slr4 protein detected in biofilm versus planktonic samples (Figure 1), suggests that Slr4 S‐layer material is a dominant component of the biofilm matrix, where it form a self‐assembling proteinaceous layer surrounding not only cells but also a wide variety of extracellular matrix components.

### 2.5 The Slr4 protein family is widespread in marine bacterial genomes and metagenomes

**FIGURE 6:**
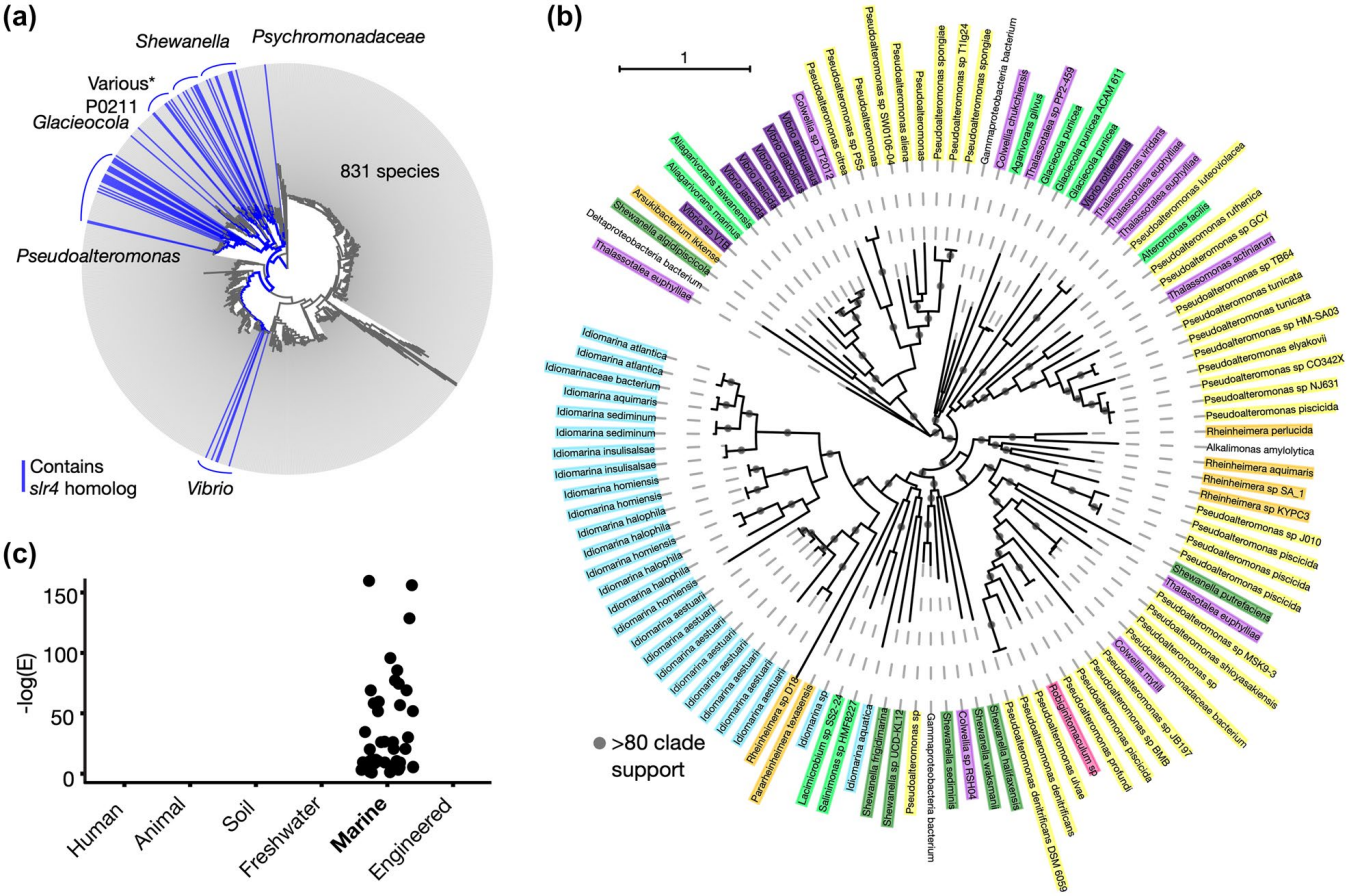
Taxonomic distribution, phylogeny, and metagenomic occurrence of Slr4 homologs. (a) Distribution of Slr4 homologs in the bacterial tree of life generated using AnnoTree. Lineages (genus‐level) containing PtSla homologs are shown in blue. Slr4 homologs were detected largely within the order Enterobacterales (GTDB taxonomy), class Gammaproteobacteria. Several instances of Slr4 were detected outside of this lineage (see Table S2). The group labeled as “Various” includes the genera *Colwelia*, *Thalassomonas*, *Thalassotalea*, and *Lacimicrobiam*. (b) Phylogenetic tree of Slr4 homologs colored according to taxonomic family. Clades with aLRT (SH‐like) branch support > 0.8 are indicated. The phylogeny was visualized using iTOL4. (c) Metagenomic survey for Slr4 homologs using the EBI MGnify metagenomics database. The plot shows the –log (*E*‐value) for all significant (phmmer *E*‐value < 0.01) Slr4 homologs detected across a range of environments. Slr4 homologs were detected exclusively in marine metagenomes

Using PSI‐BLAST, we identified 108 Slr4 homologs in available genomes, ranging in sequence identity from 13% to 60%. We then examined their distribution across the bacterial tree of life using AnnoTree (Mendler *et al*., 2019) (Figure 6a), and constructed a protein phylogeny (Figure 6b). Slr4 homologs were identified in 79 species, 19 genera, and 1 phylum. Most (98%) of these homologs (including the most closely related sequences) occur within neighboring lineages of Gammaproteobacteria, specifically within the Order Enterobacterales (GTDB nomenclature) (Figure 6a). These include additional species of *Pseudoalteromonas*, but also *Idiomarina*, *Colwellia*, *Shewanella*, *Thalassomonas*, and *Vibrio* (Table S3). According to the phylogeny, much of the Slr4 protein family has diversified consistent with taxonomy (e.g., a large Slr4 clade is specific to the genus *Idiomarina*). However, there are also examples that are inconsistent with phylogeny (e.g., Slr4 homolog from *Thalassomonas actiniarum* nested within a *Pseudoalteromonas* Slr4 clade), indicative of horizontal gene transfer (Figure 6b).

Niche analysis of Slr4‐containing species revealed that 73/79 (~92.4%) could be associated with marine habitats, and 78/79 (98.7%) with aquatic (including freshwater) habitats (Table S2). The only Slr4‐containing species not associated with aquatic environments was *Pseudoalteromonas* sp. JB197, isolated from cheese rind. A total of 27/73 (37.0%) of the marine Slr4‐containing species were found to be host‐associated (Table S2). To further explore the association with marine environments, we searched for homologs of Slr4 within available metagenomes from the EBI MGnify metagenomics resource (Mitchell *et al*., 2019) including marine, freshwater, human microbiome, soil, and engineered environments. Homologs were detected exclusively in marine metagenomes (Figure 6c).

## 3 DISCUSSION

We identified an abundant protein (EAR28894, now designated “Slr4”) produced in cultures of the marine bacterium, *P. tunicata* D2, and predicted its function as an S‐layer protein using structural bioinformatics. The purified Slr4 protein was capable of forming square (p4 symmetry) paracrystalline lattices, and these S‐layer lattices were also present and absent on the outer‐membrane of WT versus Δ*slr4* mutant cells, respectively. Consistent with the substantial increase in Slr4 relative abundance in biofilm (10%–17% of total spectra) compared to planktonic samples (2%), we observed S‐layer material shed outside of *P. tunicata* cells and identified S‐layer as a dominant component of the biofilm matrix where it is associated with OMVs and other filamentous structures. Phylogenomic and metagenomic analysis showed that Slr4‐related proteins are conserved in related species and abundant in marine Gammaproteobacteria and marine metagenomes.

### 3.1 Sequence‐to‐structure relationship

The confirmation of Slr4 as an S‐layer protein raises several questions regarding its similarity to other S‐layer protein families. Is Slr4 truly homologous to these proteins, and if so, what structural features and regions are conserved/lost between Slr4 and other S‐layer proteins? Furthermore, what sequence or structural differences account for the unique square lattice geometry of Slr4 compared to the hexagonal symmetry of RsaA (Bharat *et al*., 2017)?

Given the low sequence identity (~17% in the threading alignment) of Slr4 to the *Caulobacter* RsaA S‐layer protein, it is quite remarkable that I‐TASSER was capable of detecting this relationship and correctly predicting an S‐layer function for Slr4. Notably, a potentially significant sequence relationship could also be detected by pairwise alignment via the PredictProtein server (Yachdav *et al*., 2014); however, importantly, no relationship was detected when BLAST’s compositional bias filter was used. This suggests that I‐TASSER’s prediction of an S‐layer structure/function may have occurred in part because of a highly repetitive beta‐rich sequence composition that is shared with other beta‐rich S‐layer proteins (Baranova *et al*., 2012; Bharat *et al*., 2017). This also offers an explanation for why I‐TASSER’s threading algorithms aligned Slr4 to different regions of the RsaA template. A beta‐helical fold may represent an ideal structural solution for S‐layers due to the high propensity for self‐assembly of beta‐rich proteins (e.g., beta amyloid proteins) (Tiwari and Kepp, 2015).

Despite the distant sequence similarity between Slr4 and RsaA, previous cryo‐EM structures of RsaA (Bharat *et al*., 2017) can be used to speculate on the spatial organization of Slr4 lattices (Figure S6). Since Slr4 mapped onto a portion of the RsaA structure, it is possible that a truncated beta‐helical L‐shaped fold would result in different higher‐order interactions with its crystal form, shifting it from a hexagonal pattern (p6 symmetry) to a square grid (p4 symmetry). Based on the observed inter‐subunit spacing of ~9.1 nm which is similar to the length of the predicted Slr4 protein structure along its long axis (also ~9.1 nm), we hypothesize that four Slr4 subunits may interact within a unit cell, rather than six within the RsaA lattice. This could result in fourfold symmetric interactions, producing the square grid lattice pattern observed in TEM **(**Figures 3 and 4). This model could also provide an explanation for the “side view” of Slr4 S‐layers observed in TEM (Figure 4c), since a side view of interacting Slr4 monomers could result in apparent “V”‐shaped patterns. Future structural studies involving cryoEM and x‐ray crystallography are needed to confirm this model and further investigate the structure of the Slr4 S‐layer lattice.

### 3.2 A putative role for Slr4 in marine biofilms

Our finding that Slr4 is the dominant protein within *P. tunicata* pellicle biofilms, together with the observation of S‐layer “shedding” from cells, suggests that Slr4 may play a functional role in the biofilm matrix. Although the biological role of Slr4 in biofilms is unknown at this point, we hypothesize that it may provide protection not only to cells but also to extracellular matrix structures (e.g., filaments, OMVs, etc.) that are important for biofilm structural integrity and/or cell‐cell communication. In addition, it may provide a physical barrier shielding a larger biofilm community against external forces including predation and attack by viruses, bacteria, or eukaryotes.

A role for S‐layer proteins in biofilms is not unprecedented, as numerous studies have implicated S‐layer proteins in biofilm‐related processes such as cell adhesion to substrates, promotion of cell‐cell aggregation, and initial biofilm establishment (Beveridge *et al*., 1997; Ðapa *et al*., 2013; Janesch *et al*., 2013; Gerbino *et al*., 2015). For example, the *Bacillus subtilis* S‐layer protein (BslA) has also been shown to play a critical role in formation of pellicle biofilms (Kobayashi and Iwano, 2012; Hobley *et al*., 2013; Liu *et al*., 2017). BslA forms a hydrophobic surface layer, which aids in the formation of a biofilm at the air‐liquid interface (Kobayashi and Iwano, 2012). In addition to playing a role in the process of biofilm formation and cell‐cell aggregation, S‐layers have also been suggested to provide protective roles within biofilms, in which defenses must be heightened due to increased phage and antimicrobial attacks. A bacteriophage of *Caulobacter crescentus*, for example, is known to use the *Caulobacter* S‐layer as a receptor for invasion (Edwards and Smit, 1991). In addition, the S‐layer of *Aquaspirillum* spp. and *Aeromonas salmonicida* A449 protects the bacteria against predation by *Bdellovibrio bactervorus* (Koval and Hynes, 1991). The role of Slr4 in defense against bacteriophages and predatory bacteria will be an interesting avenue for future work.

A key finding from our bioinformatic analysis is that homologs of Slr4 were detected predominantly in Gammaproteobacteria and marine metagenomes (Figure 6a,c). Indeed, Gammaproteobacteria, including species of *Pseudoalteromonas* and *Vibrio*, are known to be particularly important members of marine biofilm communities (Mai‐Prochnow *et al*., 2004; Rao *et al*., 2005; Longford *et al*., 2007). A recent study also revealed an extraordinary level of species and functional novelty in marine microbial biofilms, with marine Gammaproteobacteria contributing the largest number of gene clusters (Zhang *et al*., 2019).

Interestingly, in addition to conservation of the Slr4 gene, the adjacent putative T2SS gene cluster was also found to be highly conserved among marine Gammaproteobacteria. This is an intriguing finding because T2S pathways are known to play a significant ecological role in marine nutrient cycling (Evans *et al*., 2008) and secretion of biofilm matrix proteins for surface colonization and biofilm development (Dang and Lovell, 2016). Thus, it is tempting to speculate that the Slr4 family of S‐layer proteins are uniquely adapted to marine microorganisms, and perhaps more specifically, marine biofilms. For instance, perhaps the structure and geometry of Slr4 S‐layers are adapted for high salinity and/or regulated exchange of marine micronutrients. Or, perhaps Slr4 provides biofilms with a level of hydrophobicity and/or cohesion that is required to withstand the constant hydrodynamic forces of marine environments.

Although previous oceanic surveys of microbial diversity have focused on planktonic microorganisms, the enormous impact of marine biofilms to biogeochemical cycling and ecosystem dynamics cannot be overstated (Zhang *et al*., 2019). Surface‐associated biofilm lifestyles provide numerous benefits to bacterial communities including resource sharing, physical support, spatial organization for enhanced nutrient acquisition and flow, and protection against phages, other bacteria, predators, antibiotics, and chemical toxins (Dang and Lovell, 2016). Cell surface associated and extracellular matrix components that protect biofilms against these agents are, therefore, fundamental elements of marine microbial ecology by providing a foundation for biofilm development.

It will be interesting to further explore the contribution of Slr4 to *P. tunicata’s* biofilm structure and physiology. It will be important to study the flow cell systems and mixed‐species biofilms under natural conditions, and examine the role of Slr4 in protecting not only cells but also biofilm matrix components against natural stressors such as antimicrobials, oxidative stress, desiccation, chemicals, temperature, and phage attack. We postulate that Slr4 plays a role in protecting both cells and biofilm matrix components against some of these stressors, thereby benefiting the fitness of marine biofilms.

## 4 EXPERIMENTAL PROCEDURES

### 4.1 Purification of S‐layer protein

*Pseudoalteromonas tunicata* strain D2 (Holmstrom *et al*., 1998) was routinely cultivated in Difco marine 2216 agar at room temperature. A 13 ml volume of the overnight‐grown culture (OD_600_ of 1.3) was subcultured into three flasks with 1 L of marine broth and incubated for 8 hr at room temperature with gentle shaking (100 rpm). Cells were harvested by centrifugation at 12,400*g* for 20 min at 4°C. The pellet was surface washed with cold phosphate‐buffered saline PBS (pH 7.4) by low centrifugation at 7,000*g* for 20 min at 4°C then resuspended in 60 ml of cold 0.1 M Tris–HCl buffer (pH 7.2). Mechanical cell disruption was applied to fragment the cell envelope (Sidhu and Olsen, 1997) and remove the capsule (Messner and Sleytr, 1987). This was achieved using a 30‐ml tissue homogenizer with a Potter‐Elvehjem PTFE pestle with six repetitions (Burden, 2008). The S‐layer was recovered by differential centrifugation; first, by pelleting the bacterial cells twice by centrifugation in a fixed angle rotor at 6,000*g* for 10 min at 4°C; and then, the supernatant was collected. The S‐layer in the supernatant was pelleted using a Beckman coulter Ti 45 fixed angle rotor ultracentrifuge at 169,645*g* for 3 hr at 4°C. The tightly packed transparent pellet was suspended in 1 ml of cold 0.1 M Tris–HCl buffer and left for 48 hr at 4°C. The suspension was put on ice with 1% v/v final concentration of RIPA solubilization buffer (0.88 g NaCl; 0.15 g EDTA; 1 g NP‐40 with a density of 1.06 g/ml; 1 g Sodium deoxycholate; 0.10 g SDS; 2.5 ml of 1 M Tris–HCl buffer, pH 7.6; and H2O to a final volume of 100 ml) for 30 min to break the non‐covalent bonds between the S‐layer and the cell wall (Beveridge, 1994; Sidhu and Olsen, 1997). The mixture was swirled occasionally for uniform spreading. A cold 0.1 M Tris–HCl buffer (pH 7.2) was added with a ratio of 3:1 of the mixture. The S‐layer was pelleted at 13,000*g* for 30 min at room temperature. The pellet was washed twice with PBS buffer (pH 7) using a 30 KDa HiPPR detergent column centrifugal filter with centrifugation at 13,000*g* for 5 min at 4ºC. The suspension was aliquoted and stored at −80°C.

### 4.2 Construction of mutant strain

#### 4.2.1 Isolation of genomic DNA

*P. tunicata* D2 was grown at 22°C for 2 days with shaking (170 rpm). Cells were collected by centrifugation and lysed with SDS and proteinase K. Genomic DNA was extracted with phenol, phenol‐chloroform and then, precipitated with ethanol. RNA was removed with RNase A. DNA was quantified using a NanoDrop spectrophotometer and checked on 0.8% agarose gel.

#### 4.2.2 Plasmid construction

One DNA fragment (1,065 bp) containing the translation start site ATG of PTD2_07619 gene was PCR amplified with primer pair JC491 and JC468 (Table S4). Another fragment (991 bp) was obtained by PCR amplification with oligos JC467 and JC492. Following gel purification, the two fragments were combined in equal amounts as PCR templates using primers JC491 and JC492. The 2 kb PCR product was restricted with BamHI and HindIII and inserted into the same sites in pK19mobsacB, yielding plasmid pJC272. The intended deletion region was Sanger sequenced with oligo JC470. All sequences are included in the supplementary information.

#### 4.2.3 Mutant construction by homologous recombination

Plasmid pJC272 was conjugated into *P. tunicata* D2 by triparental mating with the helper plasmid pRK600 (Finan *et al*., 1986). Single cross‐over recombination of the plasmid into *P. tunicata* genome was selected on marine agar with kanamycin (100 μg/ml), and streak purified on the same selection media. A single colony was grown in marine medium, diluted serially, and plated on marine agar containing 5% sucrose. Resulting clones were tested for kanamycin sensitivity (double cross‐over and loss of plasmid backbone).

#### 4.2.4 Verification of mutation

Genomic DNA was isolated from the assumed mutant. DNA fragments from wild type, the mutant, and plasmid pJC272 were PCR amplified using oligo JC470 and JC492 and resolved with 1% TAE agarose gel.

### 4.3 Biofilm growth assays

A protocol described by Robertson *et al*. (2013) with some modifications was followed to produce air‐liquid interface biofilms of *Pseudoalteromonas tunicata*. Replicate microcosms (*n* = 3) with a negative control were used. The overnight‐grown culture was diluted to OD_600_ of 1.3 and one milliliter was subcultured into 100 ml of marine broth microcosm. The cultures were incubated for 8 hr at room temperature with gentle agitation (100 rpm) then incubated statically at room temperature for 3 days. Cultures of 0 hr (8 hr planktonic cells), 26, 42, and 68 hr were inspected to observe the formation of pellicle biofilms and were imaged. Samples of pellicle biofilm were collected by aspirating the biofilm carefully from the air‐liquid interface with a sterile 10 ml serological pipette. The samples were left in the fridge for 4 hr to let the biofilm settle to the bottom of the tubes. Only 2 ml of the concentrated biofilm was collected.

#### 4.3.1 Biofilm sonication

Biofilm samples were subjected to 12 sonication cycles on ice according to a method done by Wu *et al*. (2014) with some modifications. Four probe horn tips at maximum ultrasonic frequency of 20 kHz and micro‐ultrasonic cell‐disruptor at maximum power 1,200 watts of ultrasonic energy were used. The sonicator (Kontes, Vineland, NJ) was operated at 30% (6.6 kHz) of its max amplitude. The samples were placed on ice and sonicated for 10s with rest of 50s. The protein content of the sonicated samples was measured by a NanoDrop 2000 after which the samples were sent for LC–MS/MS analysis.

### 4.4 Negative staining and image analysis

Negative staining was performed following the described procedure (Harris and De Carlo, 2014). Carbon‐coated copper grids (Ted Pella, Inc. Formvar W/CARB on 200 M CU; Prod No. 01801) were used with 2.5% w/v ammonium molybdate, pH 5‐7. A Philips CM10 microscope running at 60.0 kV was used. TEM micrograph images were analyzed using imageJ (Schneider *et al*., 2012). To measure inter‐unit spacings, five separate square grids of 15 x 15 particles were identified and the average center‐to‐center distance was calculated for each axis.

### 4.5 Analysis of Slr4 phylogenomic and metagenomic distribution

Homologs of EAR28894.1 were detected using three iterations of PSI‐BLAST (*E*‐value < 0.0001). A sequence alignment was generated using MUSCLE v3.8.31 (Edgar, 2004), and further filtered to remove partial and poorly aligning sequences. A PhyML tree (LG model) as implemented in Seaview (Gouy *et al*., 2010) was constructed from conserved alignment regions and visualized using ITOL (Letunic and Bork, 2019). The taxonomy of the aligned sequences was used to highlight the bacterial tree of life in AnnoTree (Mendler *et al*., 2019). A metagenomic survey for Slr4 homologs was performed using the EBI MGnify sequence search at https://www.ebi.ac.uk/metagenomics with default settings on May 2, 2019.

## AUTHOR CONTRIBUTIONS

A.C.D. conceived and supervised the study. S.A. and J.C. performed experiments. S.A., B.L, A.C.D., and B.M. performed data analysis. All authors interpreted findings and helped with manuscript preparation.

## Supporting information

Supplementary Material

## Data Availability

The data that supports the findings of this study are available in the supplementary material of this article, in the NCBI GenBank database (https://www.ncbi.nlm.nih.gov/protein/EAR28894), as well as in “figshare” at https://doi.org/10.6084/m9.figshare.11993505.v1.
