# Supplementary material for: Slr4, a newly identified S‐layer protein from marine Gammaproteobacteria, is a major biofilm matrix component
Source: Mol Microbiol. 2020 Sep 15;114(6):979–90. doi: 10.1111/mmi.14588 (PMC7821379; doi:10.1111/mmi.14588)
Supplement: Supplementary file 1 — Supplementary Material [file MMI-114-979-s001.pdf]

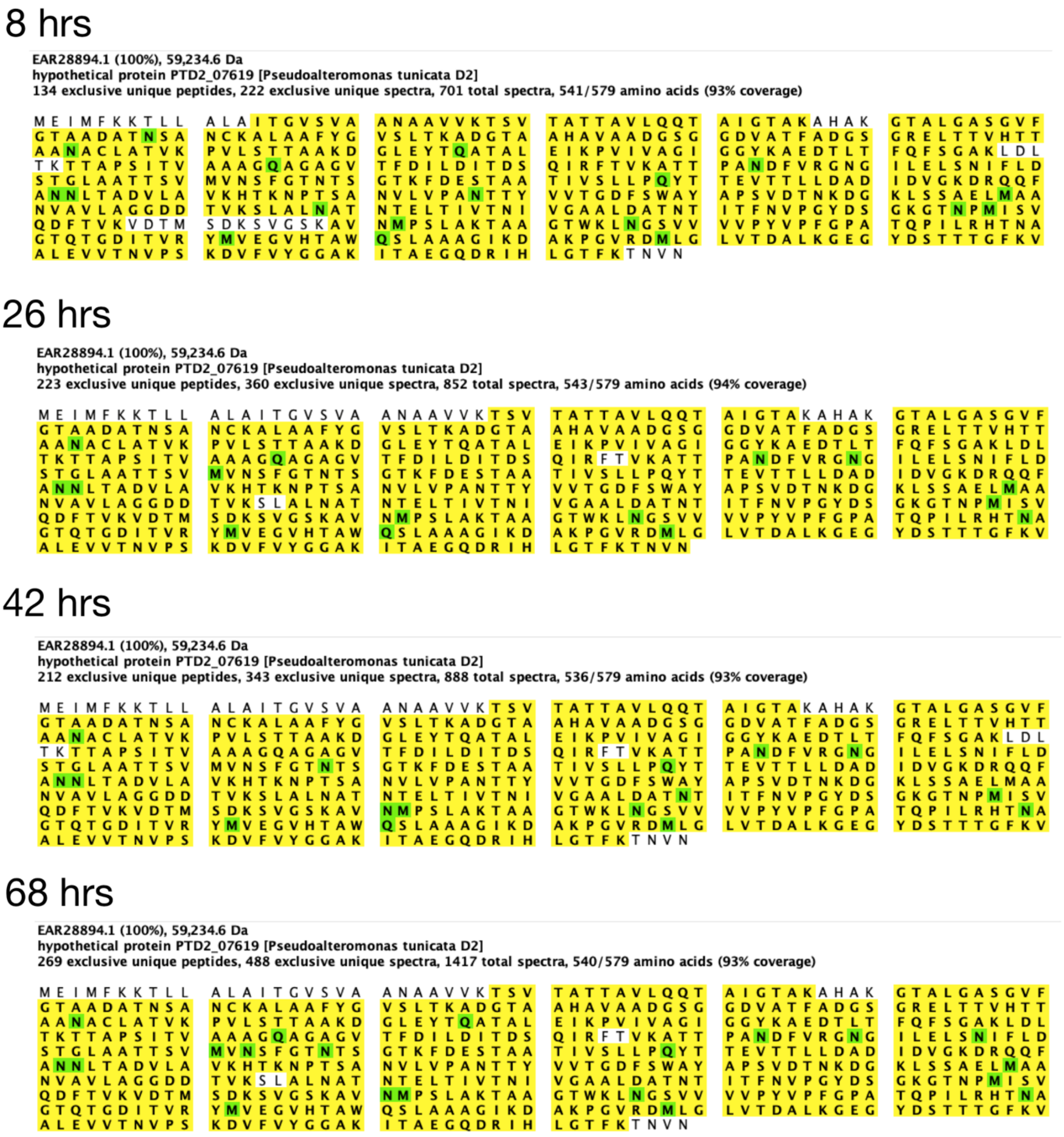

Figure S1. LC-MS/MS coverage of the EAR28894 protein. Identified peptides mapped across the entirety of the sequence, with the exception of an N-terminal segment consisting of the first 13-27 amino acid residues. Residues highlighted in green represent chemical modifications (e.g., deamidation, oxidation).

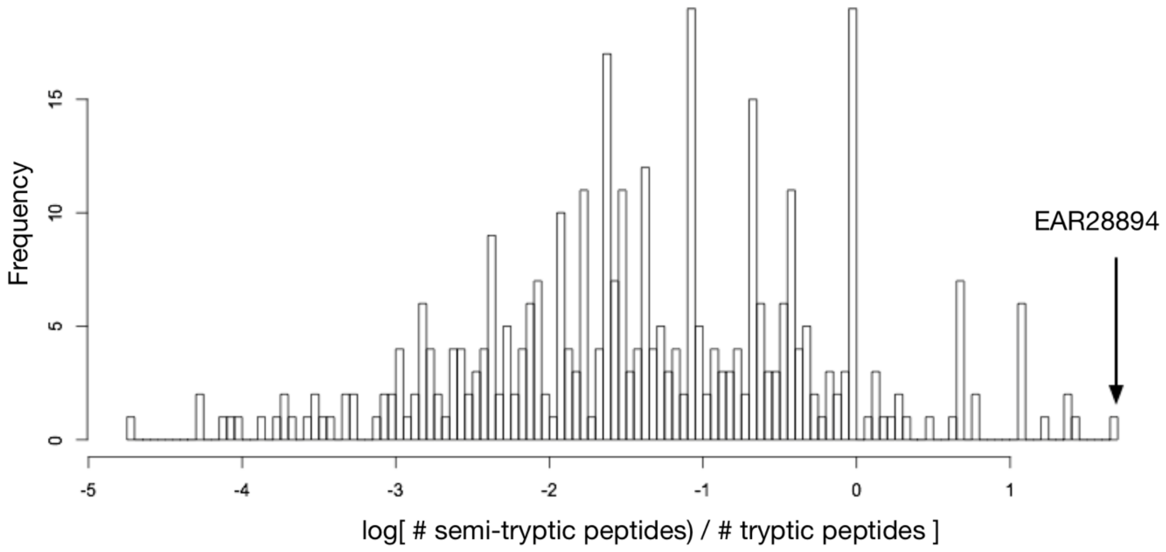

Figure S2. Ratio of semi-tryptic to tryptic peptides across all proteins in LC-MS/MS data. EAR28894 deviates from all other proteins in this regard, and therefore is likely to be most heavily processed by non-trypsin proteases.

WT Δ*slr4*

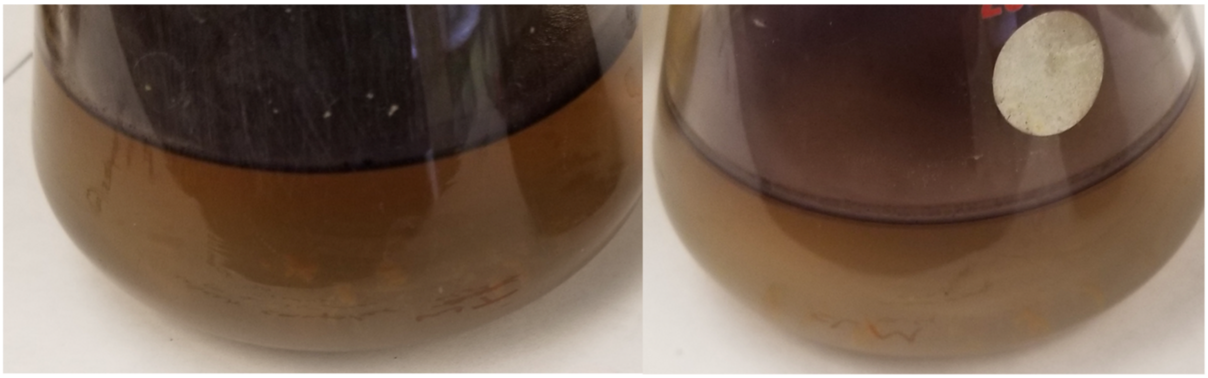

Figure S3. Pellicle biofilms formed by the WT vs Δ*slr4* mutant strain. Liquid cultures were grown for 8 hours (shaking) in liquid Difco marine broth media, followed by 72 hours of static (non-shaking) incubation.

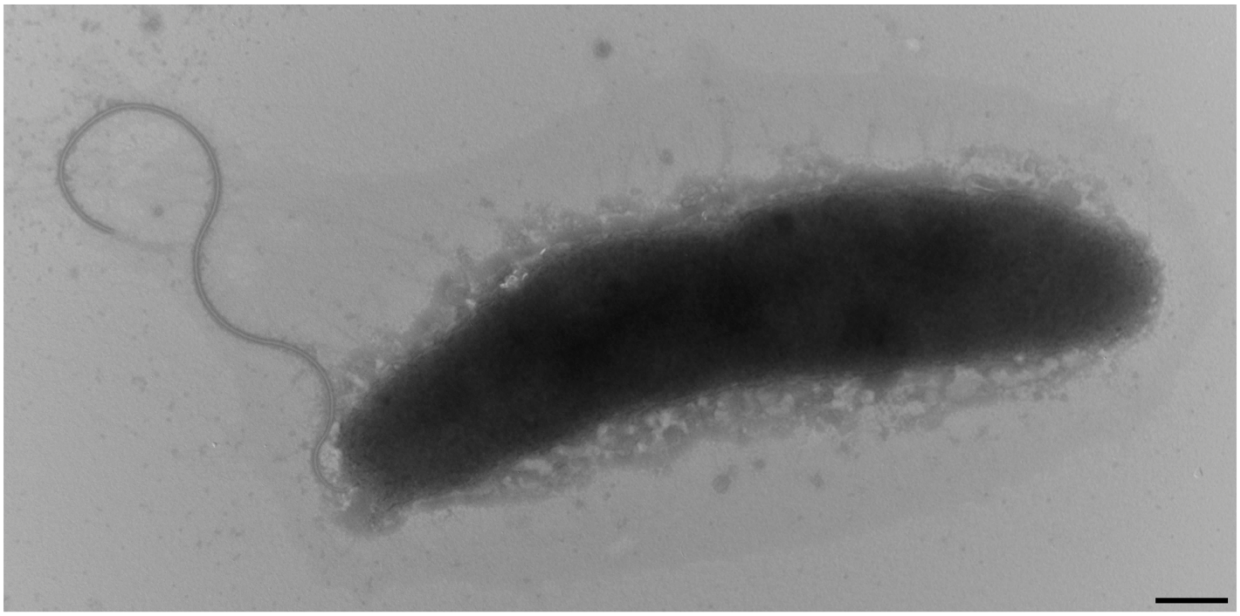

Figure S4. TEM micrograph of a *P. tunicata* cell revealing the presence of a putative outermost capsular layer. Scale bar = 500 nm.

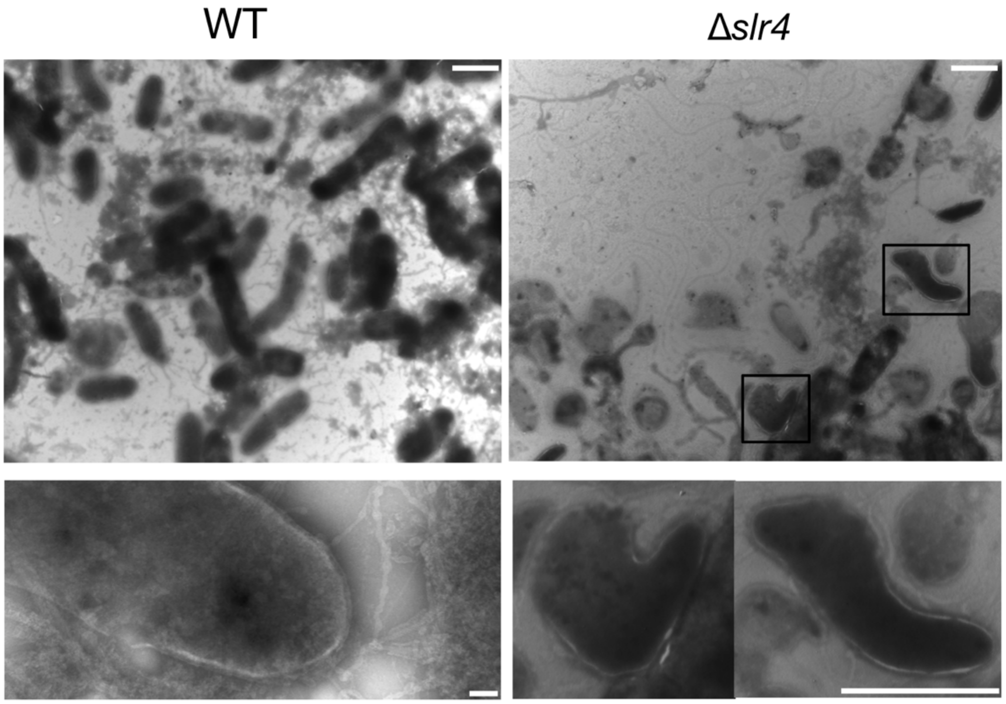

Figure S5. TEM micrographs of 48-hr pellicle biofilms (WT versus Δ*slr4* mutant strain). Top left – dense cell clusters in the WT strain interconnected by biofilm matrix material. Bottom left – a WT cell within a biofilm with extracellular biofilm matrix including fibrous structures coated by S-layer material. Top right – image taken from a Δ*slr4* biofilm showing a sparser cell distribution that is not connected by S-layer associated matrix material. Bottom right – two examples from the image above of deformed cell shapes. Scale bars are 2 microns, with the exception of the bottom left image where it is 100 nm.

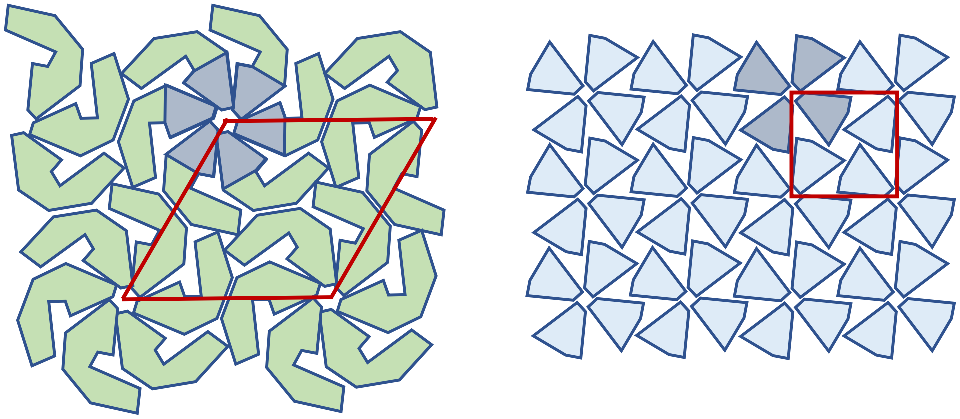

Figure S6. Hypothetical model of Slr4 S-layer lattice assembly (right) compared to rsaA S-layer assembly (left). A smaller (truncated) S-layer subunit relative to rsaA could alter inter-subunit interactions to result in a four-fold symmetric pattern (right) instead of a six-fold hexagonal symmetry (left). The model is consistent with the top-down view of a square lattice as observed in Figs. 3 and 4, as well as the side-view showing V-shaped cups as shown in Fig. 4.

Table S1. Top proteins identified by LC-MS/MS.

Accession	-10lgP	Cov. (%)	#Peptides	#Unique	PTM	Avg. Mass (kDa)	Description
**EAR28894.1**	**468.71**	**93**	**111**	**110**	**Y**	**59235**	**hypothetical protein PTD2_07619**
EAR29208.1	170.89	20	12	12	Y	68830	hypothetical protein PTD2_09189
EAR29563.1	133.95	8	3	3	Y	60109	flagellin
EAR27917.1	104.01	5	4	4	N	103265	TonB-dependent outer membrane receptor
EAR28105.1	75.5	6	3	3	N	61087	30S ribosomal subunit protein S1
EAR30644.1	56.2	0	2	2	Y	1392238	fibronectin type III domain protein
EAR27255.1	53.86	2	2	1	N	67577	putative lipoprotein
EAR28194.1	50.54	3	2	2	N	82187	TonB-dependent receptor
EAR26551.1	48.39	3	2	2	Y	105969	sensor histidine kinase/response regulator
EAR27983.1	44.82	3	1	1	N	63152	putative orphan protein

Table S2. Supporting peptides for EAR28894 identification by LC-MS/MS.

Peptide	-10lgP	Mass	L	ppm	m/z	z	RT	F	Scan	#Spec	Start	End	PTM
R.YMVEGVHTAWQSLAAAGIK.D	119.18	2031.025	19	-7.4	1016.512	2	20.76	2	3156	9	491	509	
K.DGLEYTQATALEIKPVIVAGIGGYK.A	117.52	2605.4	25	-8.2	1303.697	2	22.33	2	3432	45	140	164	
R.YM(+15.99)VEGVHTAWQSLAAAGIK.D	111.23	2047.02	19	-5.9	683.3431	3	20.02	2	3018	5	491	509	1
R.DMLGLVTDALKGEGYDSTTTGFK.V	107.36	2418.162	23	-4.7	1210.083	2	24.78	2	3863	13	517	539	
R.HTNAGTQTGDITVR.Y	104.91	1469.722	14	-6.2	735.8639	2	11.4	2	1422	4	477	490	
K.GTNPMISVQDFTVK.V	100.89	1535.765	14	-4.9	768.8862	2	20.01	2	3017	4	413	426	
K.ADGTAAHAVAADGSGGDVATFADGSGR.E	97.12	2403.069	27	-7.9	802.0238	3	14.93	2	2057	9	86	112	
R.DM(+15.99)LGLVTDALKGEGYDSTTTGFK.V	92.65	2434.157	23	-6.5	1218.078	2	20.99	2	3197	6	517	539	1
R.QQFAN(+.98)NLTADVLAVK.H	91.34	1631.852	15	-6.5	816.9279	2	19.91	2	Ta	3	298	312	2
K.VALEVVTNVPSKDVFVYGGAK.I	90.54	2191.189	21	-7.8	1096.593	2	19.65	2	2941	18	540	560	
K.GTNPM(+15.99)ISVQDFTVK.V	88.79	1551.76	14	-6.3	776.8825	2	18.42	2	2717	3	413	426	1
A.TALEIKPVIVAGIGGYK.A	88.03	1728.019	17	-8.1	865.0095	2	19.96	2	3005	2	148	164	
R.YMVEGVHTAWQ(+.98)SLAAAGIK.D	88	2032.009	19	-3.6	1017.008	2	21.43	2	3275	3	491	509	2
K.LSSAELMAANVAVLAGGDDTVK.S	87.8	2131.083	22	-5.6	1066.543	2	22.34	2	3433	4	352	373	
K.DGLEYTQ(+.98)ATALEIKPVIVAGIGGYK.A	87.28	2606.384	25	10.3	652.61	4	22.38	2	3441	12	140	164	2
R.QQFANNLTADVLAVK.H	84.32	1630.868	15	-3.8	816.4381	2	20.18	2	3053	4	298	312	
K.TSVTATTAVLQQTAIGTAK.A	83.11	1861.016	19	-7.8	931.5078	2	18.03	2	2646	3	28	46	
R.DMLGLVTDALK.G	83.06	1174.627	11	-5.3	588.3175	2	23.81	2	3689	7	517	527	
K.AEDTLTFQFSGAK.L	82.59	1413.678	13	-4.9	707.8427	2	19.86	2	2984	31	165	177	
K.ALAAFYGVSLTK.A	82.53	1239.686	12	-6.6	620.8463	2	19.91	2	2993	2	74	85	
K.LSSAELM(+15.99)AANVAVLAGGDDTVK.S	81.88	2147.078	22	0.2	1074.546	2	19.61	2	2933	4	352	373	1
R.ELTTVHTTAANAC(+57.02)LATVKPVLSTTAAK.D	81.48	2768.474	27	-5.8	693.1218	4	16.99	2	2429	5	113	139	3
R.Q(+.98)QFANNLTADVLAVK.H	81.46	1631.852	15	-6.5	816.9279	2	20.55	2	3119	2	298	312	2
K.DRQQFANNLTADVLAVK.H	80.18	1901.996	17	-4.4	952.001	2	19.83	2	2976	4	296	312	
K.LNGSVVVVPYVPFGPATQPILR.H	79.7	2322.31	22	3.3	775.1131	3	23.76	2	3680	3	455	476	
K.LN(+.98)GSVVVVPYVPFGPATQPILR.H	79.29	2323.294	22	-4.5	775.4351	3	24.08	2	3738	7	455	476	2
K.VALEVVTNVPSK.D	78.86	1254.718	12	-3.7	419.2451	3	16.45	2	2334	13	540	551	
T.ALEIKPVIVAGIGGYK.A	77.2	1626.971	16	-5.8	543.3278	3	19.84	2	2979	4	149	164	
K.GTALGASGVFGTAADATNSANCKA.L	76.07	2154.001	24	-5.8	719.0035	3	17.55	2	2553	4	51	74	
Y.MVEGVHTAWQSLAAAGIK.D	75.15	1867.961	18	-2.8	934.9854	2	20.84	2	3171	2	492	509	
R.HTN(+.98)AGTQTGDITVR.Y	74.87	1470.706	14	-5.4	736.3564	2	11.78	2	1499	3	477	490	2
K.TTAPSITVAAAGQAGAGVTFDILDITDSQIR.F	74.7	3059.577	31	-8.3	1020.858	3	25.6	2	4007	17	183	213	
H.AVAADGSGGDVATFADGSGR.E	74.54	1779.802	20	-7.6	890.9016	2	14.95	2	2061	2	93	112	
A.ANACLATVKPVLSTTAAK.D	73.72	1757.971	18	-6.2	879.9873	2	15.75	2	2208	4	122	139	
R.IHLGTFK.T	71.1	814.4701	7	0.2	408.2424	2	14.68	2	2009	5	569	575	

K.FDESTAATIVSLLPQYTTEVTTLLDADIDVGKDR.Q	70.58	3696.862	34	-5.9	1233.287	3	30.27	2	4799	18	264	297	
K.NPTSANVLVPAN(+.98)TTYVVTGDFSWAYAPSVDTNK.D	70.2	3499.678	33	-2.1	1167.564	3	23.2	2	3585	1	316	348	2
K.LSSAELMAAN(+.98)VAVLAGGDDTVK.S	70.11	2132.067	22	-4	711.6935	3	22.34	2	3434	1	352	373	2
R.GNGILELSNIFLDSTGLAATTSVMVNSFGTNTSGTK.F	68.95	3616.793	36	-5.8	1206.598	3	30.13	2	4775	4	228	263	
N.AC(+57.02)LATVKPVLSTTAAK.D	68.72	1629.912	16	-4.3	544.3091	3	15.44	2	2147	4	124	139	3
R.QQ(+.98)FANNLTADVLAVK.H	68.34	1631.852	15	-6.5	816.9279	2	20.58	2	3125	1	298	312	2
K.AVNMPSLAKTAAGTWKLNGSVVVVPYVPFGPATQPILR.H	67.65	3949.165	38	-5.7	988.2929	4	24.14	2	3749	3	439	476	
A.TTSVMVNSFGTNTSGTK.F	67.28	1730.815	17	-7.9	866.4077	2	15.57	2	2172	2	247	263	
R.ELTTVHTTAANAC(+57.02).L	66.89	1387.64	13	-5.7	694.8234	2	12.19	2	1575	2	113	125	3
K.FDESTAATIVSLLPQ(+.98)YTTEVTTLLDADIDVGKDR.Q	65.37	3697.846	34	-2.4	1233.62	3	30.34	2	4811	5	264	297	2
R.HTNAGTQTGDITVRYMVEGVHTAWQSLAAAGIK.D	65.29	3482.737	33	-2	697.5532	5	24.95	2	3895	5	477	509	
K.SLALNATNTELTIVTN(+.98)IVGAALDATN(+.98)TITFNVPGYDSGK.G	65.16	3981.011	39	3.6	1328.016	3	33.66	2	5375	2	374	412	2
D.GTAAHAVAADGSGGDVATFADGSGR.E	64.86	2217.005	25	-4.8	740.0052	3	14.56	2	1985	4	88	112	
K.SLALNATNTELTIVTN(+.98)IVGAALDATNTITFNVPGYDSGK.G	64.42	3980.027	39	-8.4	1327.672	3	34.23	2	5471	1	374	412	2
K.NPTSANVLVPANTTYVVTGDFSWAYAPSVDTNK.D	64.3	3498.694	33	-6.7	1167.231	3	23.25	2	3594	2	316	348	
K.ATTPAN(+.98)DFVR.G	63.73	1091.525	10	-3.4	546.7678	2	13.32	2	1769	4	218	227	2
K.TAAGTWK.L	63.26	733.3759	7	-3.9	367.6938	2	10.43	2	1257	1	448	454	
K.DVFVYGGAK.I	62.78	954.481	9	-3.9	478.2459	2	15.63	2	2183	2	552	560	
K.DRQQFAN(+.98)NLTADVLAVK.H	62.56	1902.98	17	-6.8	952.4907	2	19.23	2	2867	1	296	312	2
L.GLVTDALKGEGYDSTTTGFK.V	62.38	2059.011	20	-7.4	1030.505	2	24.75	2	3857	2	520	539	
K.AVNMPSLAKTAAGTWKLN(+.98)GSVVVVPYVPFGPATQPILR.H	61.37	3950.149	38	1.1	988.5457	4	24.17	2	3755	1	439	476	2
D.GSGGDVATFADGSGR.E	60.93	1352.596	15	-7.6	677.2999	2	13.8	2	1850	2	98	112	
K.TAAGTWKLNGSVVVVPYVPFGPATQPILR.H	60.92	3037.675	29	-6.8	1013.559	3	23.78	2	3684	3	448	476	
K.GTNPMISVQ(+.98)DFTVK.V	60.66	1536.749	14	-4	769.3789	2	20.63	2	3133	2	413	426	2
K.PVIVAGIGGYK.A	60.45	1072.628	11	-7.3	537.3174	2	17.27	2	2490	2	154	164	
N.GSVVVVPYVPFGPATQPILR.H	60.23	2095.183	20	-7.2	1048.591	2	23.46	2	3629	4	457	476	
C.LATVKPVLSTTAAK.D	59.96	1398.845	14	-5.3	700.4259	2	14.29	2	1938	4	126	139	
R.HTNAGTQ(+.98)TGDITVRYMVEGVHTAWQSLAAAGIK.D	59.69	3483.721	33	-1.1	871.9364	4	24.94	2	3893	1	477	509	2
A.AGQAGAGVTFDILDITDSQIR.F	59.53	2147.086	21	-3.5	1074.546	2	23.66	2	3663	4	193	213	
K.NPTSANVLVPANTTYVVTGDFSWAYAPSVDTNKDGK.L	58.83	3798.838	36	-3.3	1267.282	3	22.42	2	3449	2	316	351	
K.VALEVVTNVPSKDVF.V	58.61	1615.882	15	-11	808.9395	2	20.41	2	3095	2	540	554	

K.SLALNATNTELTIVTNIVGAALDATNTITFNVPGYDSGK.G	58.31	3979.043	39	-7.8	1327.344	3	33.45	2	5339	5	374	412	
D.ATNTITFNVPGYDSGK.G	57.54	1683.81	16	-7.7	842.906	2	18.43	2	2718	2	397	412	
R.QQFANN(+.98)LTADVLAVK.H	57.49	1631.852	15	-3.1	544.9562	3	19.97	2	3007	1	298	312	2
N.ACLATVKPVLSTTAAK.D	57.04	1572.891	16	-7.2	787.447	2	15.05	2	2079	4	124	139	
K.AVNMPSLAK.T	56.55	929.5004	9	-5.3	310.8391	3	15.31	2	2125	3	439	447	
A.HAVAADGSGGDVATFADGSGR.E	55.12	1916.861	21	-2.9	639.9592	3	14.06	2	1896	3	92	112	
F.GPATQPILR.H	54.83	951.5501	9	-7.8	476.7786	2	13.55	2	1809	2	468	476	
V.VVPYVPFGPATQPILR.H	54.53	1752.993	16	-11	877.494	2	26.21	2	4112	1	461	476	
R.DM(+15.99)LGLVTDALK.G	54.41	1190.622	11	-3.5	596.316	2	19.54	2	2921	1	517	527	1
K.LN(+.98)GSVVVVPYVPFGPATQ(+.98)PILR.H	54.03	2324.278	22	3.9	582.079	4	24.15	2	3752	4	455	476	2
R.DMLGLVTDALKGEGYD.S	53.73	1695.803	16	-10.3	848.8998	2	25.56	2	4001	2	517	532	
V.TDALKGEGYDSTTTGFK.V	53.2	1789.837	17	-5.7	895.9207	2	24.82	2	3872	1	523	539	
L.PQYTTEVTTLLDADIDVGKDR.Q	52.08	2349.17	21	-7.6	1175.583	2	29.93	2	4741	2	277	297	
V.EGVHTAWQSLAAAGIK.D	51.6	1637.853	16	-5.4	819.9291	2	20.86	2	3175	1	494	509	
L.EVVTNVPSKDVFVYGGAK.I	51.3	1907.999	18	-6.5	955.0007	2	19.73	2	2958	2	543	560	
K.DAKPGVR.D	50.41	741.4133	7	-0.5	248.1449	3	5.63	2	415	3	510	516	
R.YMVEGVHTAWQSLAAAGIKDAKPGVR.D	50.16	2754.428	26	-4.4	689.6111	4	19.1	2	2844	2	491	516	
K.ATTPANDFVR.G	50.01	1090.541	10	-1.8	364.5202	3	14.2	2	1922	4	218	227	
V.PFGPATQPILR.H	49.77	1195.671	11	-5.3	598.8397	2	23.85	2	3698	3	466	476	
K.DRQ(+.98)QFANNLTADVLAVK.H	49.75	1902.98	17	-6.8	952.4907	2	19.29	2	2877	1	296	312	2
A.LEVVTNVPSK.D	49.66	1084.613	10	-4	543.3115	2	16.52	2	2346	1	542	551	
K.AVN(+.98)MPSLAK.T	49.63	930.4844	9	3.3	466.251	2	14.94	2	2060	1	439	447	2
K.GEGYDSTTTGFK.V	49.62	1261.546	12	-5.1	631.7772	2	13.02	2	1718	2	528	539	
N.NLTADVLAVK.H	49.32	1042.602	10	-7.3	522.3046	2	17.91	2	2623	2	303	312	
V.PYVPFGPATQPILR.H	48.9	1554.856	14	-6.9	778.4298	2	26.27	2	4122	4	463	476	
R.ELTTVHTTAAN.A	48.88	1156.572	11	-0.4	579.2932	2	11.49	2	1445	2	113	123	
K.VDTMSDK.S	48.07	794.348	7	-4	398.1797	2	7.75	2	803	2	427	433	
K.ITAEGQDR.I	47.35	888.4301	8	-0.9	445.2219	2	8.25	2	888	3	561	568	
K.LN(+.98)GSVVVVPYVPFGPA.T	46.99	1614.866	16	-8.8	808.433	2	24.49	2	3810	2	455	470	2
K.PVLSTTAAK.D	46.9	886.5123	9	-4.6	444.2614	2	11.61	2	1469	2	131	139	
R.YMVEGVHTAWQS.L	46.07	1406.629	12	-3.2	704.3195	2	17.23	2	2481	1	491	502	
K.AVNM(+15.99)PSLAKTAAGTWKLNGSVVVVPYVPFGPATQPILR.H	45.14	3965.16	38	-7.2	992.2901	4	23.28	2	3599	1	439	476	1
N.AGTQTGDITVR.Y	44.77	1117.573	11	-4.3	559.7913	2	12.11	2	1560	2	480	490	
K.TTAPSITVAAAGQ(+.98)AGAGVTFDILDITDSQIR.F	44.21	3060.562	31	5	1021.2	3	46.37	2	7513	4	183	213	2
K.AVNM(+15.99)PSLAK.T	44.14	945.4954	9	-4.3	473.7529	2	11.52	2	1451	2	439	447	1
R.YMVEGVHTAWQ(+.98)SLAAAGIKDAKPGVR.D	43.22	2755.412	26	6.1	552.093	5	19.08	2	2841	1	491	516	2

K.SLALNATNTELTIVTNIVGAALDATN(+.98)TITFN(+.98)VPGYDSGK.G	43.18	3981.011	39	3.6	1328.016	3	33.59	2	5363	1	374	412	2
T.PAN(+.98)DFVR.G	42.92	818.3922	7	1.5	410.204	2	13.36	2	1776	2	221	227	2
L.ATVKPVLSTTAAK.D	41.93	1285.761	13	-2.1	643.8862	2	14.29	2	1939	2	127	139	
T.TPANDFVR.G	41.7	918.4559	8	-7.9	460.2316	2	14.13	2	1908	1	220	227	
E.VVTNVPSKDVFVYGGAK.I	41.56	1778.957	17	-9.1	890.4775	2	19.76	2	2964	1	544	560	
V.VPYVPFGPATQPILR.H	41.44	1653.924	15	-12.5	827.959	2	26.24	2	4117	1	462	476	
G.SVVVVPYVPFGPATQPILR.H	41.16	2038.162	19	-5.9	1020.082	2	23.55	2	3645	2	458	476	
T.VKPVLSTTAAK.D	40.8	1113.676	11	-2.1	372.2318	3	11.76	2	1495	2	129	139	
V.ALEVVTNVPSKDVFVYGGAK.I	40.62	2092.12	20	-9.6	1047.057	2	19.74	2	2960	1	541	560	
K.VALEVVTN(+.98)VPSKDVFVYGGAK.I	40.6	2192.173	21	-3.4	1097.09	2	21.73	2	3326	4	540	560	2
R.GNGILELSNIFLDSTGLAATTSVM(+15.99)VNSFGTN(+.98)TSGTK.F	40.27	3633.772	36	-0.2	1817.893	2	27.88	2	4394	1	228	263	1; 2
G.GDVATFADGSGR.E	39.83	1151.521	12	-8.4	576.7628	2	13.59	2	1815	1	101	112	
D.GLEYTQATALEIKPVIVAGIGGYK.A	39.54	2490.373	24	-2.3	831.1298	3	21.76	2	3331	2	141	164	
K.LNGSVVVVPYVPFGPATQ(+.98)PILR.H	39.43	2323.294	22	1.3	581.8315	4	23.86	2	3699	1	455	476	2
M.ISVQDFTVK.V	39.06	1035.56	9	-4.1	518.7852	2	17.83	2	2609	2	418	426	
T.PANDFVR.G	37.85	817.4082	7	-3.1	409.7101	2	14.13	2	1907	1	221	227	
N.PMISVQDFTVK.V	37.77	1263.653	11	-6.5	632.8298	2	20.09	2	3035	2	416	426	
R.FTVKATTPANDFVR.G	37.52	1565.82	14	-7.5	783.9115	2	16.27	2	2300	1	214	227	
N.PM(+15.99)ISVQDFTVK.V	37.07	1279.648	11	-2.5	640.8298	2	18.61	2	2753	1	416	426	1
N.IFLDSTGLAATTSVMVNSFGTNTSGTK.F	36.89	2719.337	27	-6.2	907.4474	3	23.66	2	3662	1	237	263	
R.YM(+15.99)VEGVHTAWQS.L	35.71	1422.624	12	-6.5	712.3146	2	16.29	2	2304	1	491	502	1
K.VALEVVTNVPSKD.V	35.24	1369.745	13	-6.3	685.8755	2	16.63	2	2366	1	540	552	
R.YMVEGVHTAWQSLAA.A	35.01	1661.787	15	-6.7	831.8953	2	20.16	2	3048	2	491	505	
K.ITAEGQ(+.98)DR.I	34.15	889.4141	8	-1	445.7139	2	8.79	2	979	1	561	568	2
A.TQPILR.H	33.85	726.4388	6	-3.5	364.2254	2	12.07	2	1553	2	471	476	
K.LDLTK.T	33.84	588.3483	5	-7.5	295.1792	2	11.95	2	1529	2	178	182	
V.VVVPYVPFGPATQPILR.H	33.75	1852.061	17	-7.2	927.0311	2	26.2	2	4111	1	460	476	
K.TTAPSITVAAAGQAGAGVTFDILDITDSQ(+.98)IR.F	32.91	3060.562	31	5	1021.2	3	46.71	2	7569	3	183	213	2
A.TTPANDFVR.G	32.67	1019.504	9	-4.7	510.7567	2	13.85	2	1859	1	219	227	
R.GNGILELSNIFLDSTGLAATTSVM(+15.99)VN(+.98)SFGTNTSGTK.F	32.26	3633.772	36	-5.9	1212.257	3	27.87	2	4392	1	228	263	1; 2
A.NAC(+57.02)LATVKPVLSTTAAK.D	32.25	1743.955	17	-6	872.9797	2	15.66	2	2191	1	123	139	3
N.VAVLAGGDDTVK.S	31.78	1143.614	12	-9.1	572.8088	2	14.06	2	1895	2	362	373	
R.ELTTVHTTAANA.C	31.73	1227.61	12	-5.2	614.8088	2	12.26	2	1587	1	113	124	
D.TLTFQFSGAK.L	31.72	1098.571	10	-7.2	550.2888	2	18.03	2	2645	2	168	177	
D.VLAVK.H	31.7	528.3635	5	-3.2	265.1882	2	10.56	2	1281	1	308	312	
V.PSKDVFVYGGAK.I	31.19	1266.661	12	-11.4	634.3304	2	19.72	2	2957	1	549	560	

I.HLGTFK.T	31.06	701.386	6	-3.1	351.6992	2	14.66	2	2005	2	570	575	
R.GNGILELSNIFLDSTGLAATTSVM(+15.99)VNSFGTNTSGTK.F	30.98	3632.788	36	-7.2	1211.928	3	27.78	2	4376	1	228	263	1
K.AEDTLTFQ(+.98)FSGAK.L	30.85	1414.662	13	9.7	708.345	2	25.55	2	3999	3	165	177	2
T.TPAN(+.98)DFVR.G	29.92	919.4399	8	-1.2	460.7267	2	13.37	2	1778	1	220	227	2
V.ALEVVTNVPSK.D	29.52	1155.65	11	-1.4	578.8314	2	16.56	2	2354	2	541	551	
G.SGGDVATFADGSGR.E	28.96	1295.574	14	-11.1	648.7872	2	13.77	2	1845	1	99	112	
H.TNAGTQTGDITVR.Y	28.86	1332.663	13	-6.2	667.3348	2	12.41	2	1614	1	478	490	
R.ELTTVHTTAA.N	28.71	1042.53	10	-4.7	522.2696	2	12.13	2	1564	1	113	122	
R.DM(+15.99)LGLVTDALKGEGYD.S	27.7	1711.798	16	-8.4	856.8988	2	21.26	2	3246	1	517	532	1
K.ITAEGQDRIHLGTFK.T	27.54	1684.89	15	-4.1	422.228	4	15.54	2	2167	1	561	575	
R.YM(+15.99)VEGVHTAWQ.S	26.46	1335.592	11	-11.9	668.7952	2	16.49	2	2341	1	491	501	1
A.TVKPVLSTTAAK.D	26.23	1214.723	12	-9.6	608.3632	2	14.35	2	1949	1	128	139	
K.NPTSANVLVPANTTY.V	26.21	1560.778	15	-11.4	781.3875	2	17.88	2	2619	1	316	330	
T.NPMISVQDFTVK.V	26.19	1377.696	12	-7.5	689.8502	2	20.05	2	3026	1	415	426	
K.TSVTATTAVLQQTAIGTAKAHA.K	26.18	2140.149	22	-10	714.3831	3	17.72	2	2588	1	28	49	
K.ADGTAAHAVAAD.G	25.95	1068.484	12	-4.9	535.2464	2	9.71	2	1134	1	86	97	
D.ILDITDSQIR.F	25.55	1172.64	10	-7	587.3232	2	17.34	2	2502	1	204	213	
R.HTN(+.98)AGTQ(+.98)TGDITVR.Y	25.35	1471.69	14	-0.1	736.8524	2	12.34	2	1601	1	477	490	2
K.GTNPM(+15.99)ISVQ(+.98)DFTVK.V	23.46	1552.744	14	-2.9	777.3771	2	18.9	2	2809	1	413	426	1; 2
V.FVYGGAK.I	22.94	740.3857	7	-5.8	371.198	2	15.66	2	2190	1	554	560	
R.ELTTVHTTAAN(+.98)AC(+57.02).L	22.87	1388.624	13	8.2	695.3251	2	12.27	2	1589	1	113	125	2; 3

* L = Length; F = Fraction; 1 - Oxidation (M); 2 - Deamidation (NQ); 3 - Carbamidomethylation

Table S3. Niche analysis of species/genomes with detected EAR28894 (Slr4) homologs.

Protein sequence (accession #)	Species	Marine	Host-associated	Fresh water	Habitat description	Reference/Source
WP_055732151.1	*Agarivorans gilvus*	Yes	Yes		Seaweed	https://www.ncbi.nlm.nih.gov/pubmed/20363930
WP_026972292.1	*Aliagarivorans marinus*	Yes			Seawater	https://www.dsmz.de/catalogues/details/culture/DSM-23064.html
WP_026957844.1	*Aliagarivorans taiwanensis*	Yes			Seawater	https://www.ncbi.nlm.nih.gov/pubmed/19567569
WP_091340846.1	*Alkalimonas amylolytica*	No		Yes	Lake Chahannor in China	https://www.ncbi.nlm.nih.gov/pubmed/14986177
WP_124748942.1	*Alteromonas facilis*	Yes	Yes		Isolated from a sea cucumber culture pond in China	https://www.ncbi.nlm.nih.gov/pubmed/30528643
WP_046556214.1	*Arsukibacterium ikkense*	Yes			Isolated from the alkaline, low-saline ikaite columns in the Ikka Fjord, SW Greenland	https://www.ncbi.nlm.nih.gov/pubmed/16790334
WP_085282491.1	*Colwellia chukchiensis*	Yes			Isolated from seawater samples from the Chukchi Sea in the Arctic Ocean	https://www.ncbi.nlm.nih.gov/pubmed/20495042
WP_085298075.1	*Colwellia mytili*	Yes	Yes		Isolated from the mussel Mytilus edulis from the South Sea in Korea	https://www.ncbi.nlm.nih.gov/pubmed/27902189
WP_118961217.1	*Colwellia sp. RSH04*	Yes			Seawater	https://www.ebi.ac.uk/biosamples/samples/SAMN09916314
WP_057830656.1	*Colwellia sp. TT2012*	Yes			Marine sediment, Pacific Ocean: the Tonga Trench	https://www.ncbi.nlm.nih.gov/nuccore/NZ_LJYX00000000.1
RLB69876.1	*Deltaproteobacteria bacterium*	Yes			marine sediment metagenome; deep-sea hydrothermal vent sediments from dive 4571_4 depth 0-3 cm	https://www.ncbi.nlm.nih.gov/protein/RLB69876.1
PCH94196.1,PCI59289.1	*Gammaproteobacteria bacterium*	Yes			mediterranean seawater-France	https://www.uniprot.org/taxonomy/1805126
WP_040521162.1, WP_070111740.1	*Glaciecola punicea*	Yes			isolated from sea-ice cores collected from coastal areas of eastern Antarctica	https://ijs.microbiologyresearch.org/content/journal/ijsem/10.1099/00207713-48-4-1213
GAB56998.1	*Glaciecola punicea ACAM 611*	Yes			Antarctic sea ice	https://jb.asm.org/content/194/12/3267
PTB83247.1,PTB83248.1,PTB84530.1,PTB85125.1,PTB85126.1,RUO41646.1,RUO41647.1,RUO41648.1	*Idiomarina aestuarii*	Yes			isolation of the type strain from shallow coastal seawater.	https://ijs.microbiologyresearch.org/content/journal/ijsem/10.1099/ijs.0.022970-0
WP_133538702.1	*Idiomarina aquatica*	Yes			Isolated from salterns	https://ijs.microbiologyresearch.org/content/journal/ijsem/10.1099/ijsem.0.000619
WP_126834469.1	*Idiomarina aquimaris*	Yes	Yes		isolated from the reef-building coral Isopora palifera	https://ijs.microbiologyresearch.org/content/journal/ijsem/10.1099/ijs.0.035592-0
KFZ29179.1,WP_034731676.1	*Idiomarina atlantica*	Yes			isolated from the deep sea sediment of the North Atlantic Ocean	https://link.springer.com/article/10.1007%2Fs10482-014-0337-7

RUO53158.1,WP_126763865.1,WP_126763868.1,WP_126763869.1	*Idiomarina halophila*	Yes			isolated from the sediment of the solar saltern pond located in Gomso, Republic of Korea	https://www.microbiologyresearch.org/docserver/fulltext/ijsem/65/4/1268_ijs000094.pdf
RUO56263.1,WP_126771356.1,WP_126771358.1,WP_126771362.1	*Idiomarina homiensis*	Yes			isolated from seashore sand in Korea	https://www.microbiologyresearch.org/docserver/fulltext/ijsem/56/9/2229.pdf
RUO62606.1,WP_126753970.1	*Idiomarina insulisalsae*	Yes			isolated from a sea salt evaporation pond on the Island of Sal in the Cape Verde Archipelago	https://pdf.sciencedirectassets.com/273235/1-s2.0-S0723202009X00068/1-s2.0-S072320200900085X/main.pdf
WP_026861598.1, WP_126775152.1	*Idiomarina sediminum*	Yes			isolated from the soil of a sea salt evaporation pond	https://www.microbiologyresearch.org/docserver/fulltext/ijsem/60/1/1.pdf
HAD47721.1	*Idiomarina sp.*	Yes			marine metagenome	https://www.ncbi.nlm.nih.gov/pubmed/30148503
MBG23301.1	*Idiomarinaceae bacterium*	Yes			marine metagenome from a Mediterranean Sea water sample	https://www.ncbi.nlm.nih.gov/pubmed/29337314
WP_088331539.1	*Lacimicrobium sp. SS2-24*	Yes	Yes		sediment from sea cucumber culture	https://www.ncbi.nlm.nih.gov/nuccore/NZ_NIWW00000000.1
WP_031571199.1	*Pararheinheimera texasensis*	No		Yes	USA: Spring Lake; San Marcos; Texas	http://gtdb.ecogenomic.org/genomes?gid=GCF_000711985.1
MBU77382.1	*Pseudoalteromonadaceae bacterium*	Yes			marine metagenome from a South Atlantic Ocean water sample	https://www.ncbi.nlm.nih.gov/pubmed/29337314
WP_077536094.1	*Pseudoalteromonas aliena*	Yes			Sea of Japan, Pacific Ocean	https://www.ncbi.nlm.nih.gov/pubmed/15388692
WP_010361438.1	*Pseudoalteromonas citrea*	Yes			Marine	https://www.ncbi.nlm.nih.gov/pubmed/12190022/
WP_091983032.1	*Pseudoalteromonas denitrificans*	Yes			Marine	https://doi.org/10.1248/bpb.22.532
SFC52264.1	*Pseudoalteromonas denitrificans DSM 6059*	Yes			Marine	https://www.ncbi.nlm.nih.gov/Taxonomy/Browser/wwwtax.cgi?lvl=0&id=43656, https://www.microbiologyresearch.org/docserver/fulltext/ijsem/37/4/ijs-37-4-416.pdf
KID36130.1	*Pseudoalteromonas elyakovii*	Yes			isolated from marine environments	https://www.microbiologyresearch.org/docserver/fulltext/ijsem/53/1/125.pdf
CCQ10312.1	*Pseudoalteromonas luteoviolacea B = ATCC 29581*	Yes			Marine	https://www.ncbi.nlm.nih.gov/pmc/articles/PMC3593315/
WP_010368594.1, WP_045962959.1, WP_045987911.1, WP_088531627.1, WP_117332793.1	*Pseudoalteromonas piscicida*	Yes			found in the marine environment	https://www.ncbi.nlm.nih.gov/pubmed/28363962
WP_119852593.1	*Pseudoalteromonas profundi*	Yes			isolated from a deep-sea seamount	https://www.ncbi.nlm.nih.gov/pubmed/27493151
WP_022946007.1	*Pseudoalteromonas ruthenica*	Yes	Yes		isolated from marine invertebrates	https://www.microbiologyresearch.org/docserver/fulltext/ijsem/52/1/0520235a.pdf
WP_130050461.1	*Pseudoalteromonas shioyasakiensis*	Yes			isolated from Pacific Ocean sediment	https://www.ncbi.nlm.nih.gov/pubmed/24021728
MAD02172.1	*Pseudoalteromonas sp.*	Yes			marine metagenome from a Pacific Ocean water sample	https://www.ncbi.nlm.nih.gov/pubmed/29337314
MBD58444.1	*Pseudoalteromonas sp.*	Yes			marine metagenome from an Indian Ocean water sample	https://www.ncbi.nlm.nih.gov/pubmed/29337314

WP_042150686.1	*Pseudoalteromonas sp. '520P1 No. 412'*	Yes		Marine	https://mra.asm.org/content/2/6/e01346-14
WP_042150686.1	*Pseudoalteromonas sp. '520P1 No. 423'*	Yes		Marine	https://mra.asm.org/content/2/6/e01346-14
WP_069020791.1	*Pseudoalteromonas sp. BMB*	Yes	Yes	Isolated from gut of comb jelly	https://www.uniprot.org/proteomes/UP000094131
WP_130151616.1	*Pseudoalteromonas sp. CO133X*	Yes	Yes	Octocoral	https://onlinelibrary.wiley.com/doi/full/10.1002/jobm.201800087
WP_130151616.1	*Pseudoalteromonas sp. CO302Y*	Yes	Yes	Octocoral	https://onlinelibrary.wiley.com/doi/full/10.1002/jobm.201800087
WP_130126916.1	*Pseudoalteromonas sp. CO342X*	Yes		host-associated (Muricea sp.), Panama: Coiba National Park	https://www.ncbi.nlm.nih.gov/nuccore/1577351741
WP_099029058.1	*Pseudoalteromonas sp. GCY*	Yes		Isolated from Northern Yellow Sea	https://www.ncbi.nlm.nih.gov/nuccore/NZ_NHNM00000000.1
WP_095726933.1	*Pseudoalteromonas sp. HM-SA03*	Yes	Yes	Source (marine), Host (Hapalochlaena), Location (Australia: Moreton Bay)	https://www.ncbi.nlm.nih.gov/nuccore/NZ_NSDG00000000.1
WP_125251093.1	*Pseudoalteromonas sp. J010*	Yes	Yes	Isolated from surface of crustose coralline alga	https://www.ncbi.nlm.nih.gov/nuccore/NZ_RDBW00000000.1
WP_086997608.1	*Pseudoalteromonas sp. JB197*	No		Isolated from cheese rind	https://www.ncbi.nlm.nih.gov/nuccore/NZ_NRGZ00000000.1
WP_119861005.1	*Pseudoalteromonas sp. MSK9-3*	Yes		Isolated from surface saline water	https://www.ncbi.nlm.nih.gov/nuccore/NZ_MIET00000000.1
WP_017217139.1	*Pseudoalteromonas sp. NJ631*	Yes	Yes	Isolation source (ocean), host - Hymeniacidon perleve (marine sponge)	https://www.ncbi.nlm.nih.gov/nuccore/NZ_AKXJ00000000.1
WP_128731076.1	*Pseudoalteromonas sp. PS5*	Yes	Yes	Isolated from (host: Neogoniolithon solubile), USA: reef near Looe Key	https://www.ncbi.nlm.nih.gov/nuccore/NZ_RCSQ00000000.1
WP_053910113.1	*Pseudoalteromonas sp. SW0106-04*	Yes		Seawater (Indian Ocean)	https://www.ebi.ac.uk/biosamples/samples/SAMD00039894

WP_105171055.1	*Pseudoalteromonas sp. T1lg24*	Yes			Isolated from sea water (sediment depth)	https://www.ncbi.nlm.nih.gov/nuccore/NZ_PQCB00000000.1
WP_024611180.1	*Pseudoalteromonas sp. TB64*	Yes	Yes		Isolated from a sponge, Antarctica	https://www.ncbi.nlm.nih.gov/nuccore/NZ_AUTQ00000000.1
WP_010561144.1, WP_100912554.1	*Pseudoalteromonas spongiae*	No	Yes	Yes	isolated from the surface of the sponge Mycale adhaerens in Hong Kong waters	https://www.ncbi.nlm.nih.gov/pubmed/16014487
WP_009838156.1, WP_119081635.1	*Pseudoalteromonas tunicata*	Yes			Marine organism originally isolated from tunicates	https://www.ncbi.nlm.nih.gov/pubmed/9828422
WP_086744222.1	*Pseudoalteromonas ulvae*	Yes	Yes		Isolated from surface of marine alga	https://www.ncbi.nlm.nih.gov/pubmed/11491351
WP_134053939.1	*Rheinheimera aquimaris*	Yes			isolated from seawater of the East Sea in Korea	https://www.ncbi.nlm.nih.gov/pubmed/17625162
WP_019674711.1	*Rheinheimera perlucida*	Yes			Isolated from surface water from Baltic Sea	https://www.ncbi.nlm.nih.gov/pubmed/16957117
WP_132584183.1	*Rheinheimera sp. D18*	Yes			Isolated from yellow sea (China)	https://www.ncbi.nlm.nih.gov/nuccore/NZ_CP037745.1
WP_127697655.1	*Rheinheimera sp. KYPC3*	No		Yes	Isolated from freshwater stream	http://link-springer-com-443.webvpn.jxust.edu.cn/article/10.1007%2Fs00203-019-01657-5
WP_068063761.1	*Rheinheimera sp. SA_1*	No		Yes	isolated from "iron backwash sludge of a waterworks in Germany"	https://www.ncbi.nlm.nih.gov/pmc/articles/PMC4991719/

PHS21107.1	*Robiginitomaculum sp.*	Yes		from Antarctic seawater	http://www.bacterio.net/robiginitomaculum.html
WP_109338879.1	*Salinimonas sp. HMF8227*	Yes		Isolated from saltern in South Korea	https://www.ncbi.nlm.nih.gov/nuccore/NZ_CP029347.1
WP_119977519.1	*Shewanella algidipiscicola*	Yes	Yes	isolated from marine fish of the Danish Baltic Sea	https://www.ncbi.nlm.nih.gov/pubmed/17267977
WP_059744161.1	*Shewanella frigidimarina*	Yes		Isolated from Antarctic coastal areas	https://www.ncbi.nlm.nih.gov/pubmed/11837303
WP_108947122.1	*Shewanella halifaxensis*	Yes		Isolated from marine sediment	https://www.ncbi.nlm.nih.gov/pubmed/16403888
WP_115405978.1	*Shewanella putrefaciens*	Yes	Yes	Associated with spoiled fish	https://www.ncbi.nlm.nih.gov/pubmed/2641275
WP_012143353.1	*Shewanella sediminis*	Yes		Isolated from marine sediment	https://www.ncbi.nlm.nih.gov/pubmed/16014474
WP_076411007.1	*Shewanella sp. UCD-KL12*	Yes	Yes	Isolated from seagrass	https://www.ncbi.nlm.nih.gov/pubmed/28360178
WP_028771745.1	*Shewanella waksmanii*	Yes	Yes	Marine strain isolated from sipuncula	https://www.ncbi.nlm.nih.gov/pubmed/13130035
WP_044830703.1	*Thalassomonas actiniarum*	Yes	Yes	Isolated from marine animals	https://www.ncbi.nlm.nih.gov/pubmed/19329588/
WP_044836570.1	*Thalassomonas viridans*	Yes		isolated from oysters off the Mediterranean coast	https://www.ncbi.nlm.nih.gov/pubmed/11491324
WP_115999569.1, WP_116007234.1, WP_116007235.1, WP_116014431.1	*Thalassotalea euphylliae*	Yes	Yes	Isolated from coral	https://www.ncbi.nlm.nih.gov/pubmed/27582443
WP_074500610.1	*Thalassotalea sp. PP2-459*	Yes	Yes	Isolated from clam larvae in shellfish hatchery in Spain	https://www.ncbi.nlm.nih.gov/pubmed/23743010

WP_074191941.1	*Vibrio antiquarius*	Yes		Isolated from deep sea hydrothermal vent	https://www.pnas.org/content/112/21/E2813
WP_104968991.1	*Vibrio diabolicus*	Yes	Yes	Isolated from a deep-sea hydrothermal vent annelid worm	https://www.ncbi.nlm.nih.gov/pubmed/9336897
WP_005434363.1	*Vibrio harveyi*	Yes	Yes	Pathogen of marine vertebrates and invertebrates	https://onlinelibrary.wiley.com/doi/abs/10.1111/j.1472-765X.2006.01989.x
WP_038864853.1, WP_045422135.1	*Vibrio jasicida*	Yes	Yes	Isolated from marine vertebrates and invertebrates	https://www.ncbi.nlm.nih.gov/pubmed/21984666
WP_088881413.1	*Vibrio rotiferianus*	Yes	Yes	Marine pathogen	https://www.ncbi.nlm.nih.gov/pmc/articles/PMC3133291/
WP_095760150.1	*Vibrio sp. V1B*	Yes	Yes	Isolated from gut of saltwater clam	https://www.ncbi.nlm.nih.gov/pubmed/29051252

Table S4. Oligos used for plasmid construction

Oligo ID	Sequence
JC467	TCAGTTTCATGAAACTTGGAGAATGCTGCAGTAAGCAACCGCTAAGTTTAAGTAGT
JC468	ACTACTTAAACTTAGCGGTTGCTTACTGCAGCATTCTCCAAGTTTCATGAAACTGA
JC470	CGGCAGGCTTAAACTTGCTGCGTT
JC491	GCGGGATCCAAACAATTATTTTAGGTGGTTTAATTAG
JC492	GCGCAAGCTTGCCGATTAATGGACCAAGTGTCAAC

Supplementary Text

>Protein PTD2_07619 (EAR28894)
MEIMFKKTLLALAITGVSVAANAAVVKTSVTATTAVLQQTAIGTAKAHAKGTALGASGVFGTAADATNSANCKALAA
FYGVSLTKADGTAAHAVAADGSGGDVATFADGSGRELTTVHTTAANACLATVKPVLSTTAAKDGLEYTQATALEIKP
VIVAGIGGYKAEDTLTFQFSGAKLDLTKTTAPSITVAAAGQAGAGVTFDILDITDSQIRFTVKATTPANDFVRGNGI
LELSNIFLDSTGLAATTSVMVNSFGTNTSGTKFDESTAATIVSLLPQYTTEVTTLLDADIDVGKDRQQFANNLTADV
LAVKHTKNPTSANVLVPANTTYVVTGDFSWAYAPSVDTNKDGKLSSAELMAANVAVLAGGDDTVKSLALNATNTELT
IVTNIVGAALDATNTITFNVPGYDSGKGTNPMISVQDFTVKVDTMSDKSVGSKAVNMPSLAKTAAGTWKLNGSVVVV
PYVPFGPATQPILRHTNAGTQTGDITVRYMVEGVHTAWQSLAAAGIKDAKPGVRDMLGLVTDALKGEGYDSTTTGFK
VALEVVTNVPSKDVFVYGGAKITAEGQDRIHLGTFKTNVN

>PTD2_07619 (EAR28894) deletion/replacement
MHQ

>**PTD2_07619 (EAR28894) gene**
ATGGAAATTATGTTCAAGAAAACTCTACTAGCACTAGCAATTACTGGTGTTTCTGTAGCAGCTAATGCAGCTGTGGT
AAAAACAAGCGTAACAGCAACTACTGCTGTTTTACAACAAACAGCAATTGGTACTGCTAAAGCTCACGCTAAAGGTA
CTGCTCTAGGTGCTTCTGGTGTGTTCGGTACAGCTGCAGATGCAACAAATTCAGCTAACTGTAAAGCATTAGCTGCT
TTTTATGGTGTTTCATTAACTAAAGCTGATGGTACAGCTGCTCACGCTGTTGCTGCTGATGGTTCAGGTGGCGACGT
AGCTACTTTTGCTGATGGTTCAGGTCGTGAATTAACAACAGTACACACTACAGCTGCTAATGCTTGTCTTGCAACTG
TTAAACCAGTTCTTTCTACTACCGCTGCTAAAGATGGTCTTGAGTACACTCAAGCTACAGCACTTGAAATTAAACCA
GTTATTGTTGCTGGTATCGGTGGTTACAAAGCTGAAGATACGTTAACGTTCCAATTCTCTGGCGCTAAATTAGATTT
AACTAAAACAACTGCTCCATCTATTACTGTTGCAGCAGCTGGTCAAGCTGGTGCTGGTGTGACATTTGATATCCTTG
ATATCACTGATTCACAAATTCGTTTCACCGTTAAAGCAACTACTCCAGCGAATGATTTTGTTCGTGGTAACGGTATT
TTAGAATTAAGCAACATCTTCCTAGATTCTACAGGTCTTGCTGCTACTACTTCTGTAATGGTTAATTCTTTTGGTAC
TAATACATCAGGTACTAAATTTGATGAATCAACAGCTGCAACGATTGTATCTTTACTTCCACAATACACTACAGAAG
TAACTACGTTACTTGATGCAGATATCGATGTAGGTAAAGATCGTCAACAATTTGCTAACAACTTAACAGCTGATGTT
TTAGCTGTTAAACACACTAAAAACCCAACATCTGCTAACGTATTAGTTCCTGCTAATACAACTTATGTTGTTACTGG
TGATTTCTCTTGGGCTTATGCGCCAAGCGTAGATACAAACAAAGATGGCAAATTATCTTCAGCTGAGTTAATGGCTG
CGAATGTTGCTGTTTTAGCTGGTGGTGATGATACAGTTAAGTCTTTAGCTCTTAATGCTACAAACACTGAATTAACA
ATTGTTACTAATATTGTTGGTGCTGCATTAGATGCAACTAACACTATTACATTCAATGTTCCAGGCTATGATTCAGG
TAAAGGCACTAACCCAATGATTTCTGTTCAAGATTTCACGGTTAAAGTTGATACTATGTCTGATAAATCAGTTGGTA
GCAAAGCTGTAAACATGCCATCACTTGCTAAAACAGCAGCTGGTACATGGAAATTAAATGGTTCTGTTGTTGTTGTT
CCTTATGTTCCATTTGGTCCAGCTACACAACCAATTTTACGCCACACTAATGCTGGTACACAAACTGGTGATATCAC
AGTTCGTTACATGGTTGAAGGTGTTCACACTGCATGGCAATCACTTGCAGCAGCTGGCATCAAAGATGCAAAACCTG
GCGTACGTGATATGTTAGGCCTAGTAACTGACGCACTTAAAGGTGAAGGTTATGACTCTACAACTACTGGCTTCAAA
GTAGCTTTAGAAGTTGTAACTAACGTTCCTTCAAAAGACGTGTTCGTATACGGCGGCGCTAAAATCACTGCTGAAGG
TCAAGATCGTATCCATTTAGGTACTTTCAAAACTAACGTAAACTAA

> PTD2_07619 (EAR28894) deletion/replacement
ATGCTGCAGTAA
